# Comparative clinical outcomes of acenocoumarol versus direct oral anticoagulants (DOACs) and warfarin in patients with atrial fibrillation: real-world-evidence (SIESTA-A study)

**DOI:** 10.3389/fphar.2025.1548298

**Published:** 2025-08-01

**Authors:** Ma Carmen Montero-Balosa, Juan A. Limón-Mora, Ana Leal-Atienza, Beatriz García-Robredo, Pablo Sánchez-Villegas, Rebeca Isabel-Gómez, Ma José Aguado-Romeo, Luis Gabriel Luque Romero, Ma Teresa Molina-López

**Affiliations:** ^1^ Primary Care Pharmacy, Aljarafe-Sevilla Norte District. Andalusian Health Service, Seville, Spain; ^2^ Coordination of Management and Evaluation, Andalusian Health Service., Seville, Spain; ^3^ Andalusian Public Foundation for the Management of Health Research in Seville (FISEVI), Seville, Spain; ^4^ Primary Care Pharmacy, Seville District. Andalusian Health Service, Seville, Spain; ^5^ Andalusian School of Public Health, Granada, Spain; ^6^ Andalusian Health Technology Assessment Area, Progress and Health Foundation, Seville, Spain; ^7^ Center for the Transfusion of Tissues and Cells, Seville, Spain; ^8^ Research Unit, Andalusian Health Service, Seville, Spain

**Keywords:** oral anticoagulant agents, oral direct anticoagulants, safety, health outcomes, effectiveness

## Abstract

**Objective:**

The aim of this study was to evaluate the effectiveness and safety of direct oral anticoagulants (DOACs: dabigatran, rivaroxaban, apixaban and edoxaban) and warfarin versus acenocoumarol in patients with atrial fibrillation under real-world clinical practice conditions.

**Methods:**

This was a retrospective, real-world data-based study. The data source was the Andalusian Population Health Database. The study covered the period from January 2012 to December 2020. Effectiveness outcomes were defined as the identification of a first occurrence of ischaemic or bleeding events, or all-cause mortality. The statistical analysis included crude incidence analysis, survival models: Kaplan-Meier curves, propensity score matched pairs analysis, Fine-Gray model, and Cox regression analysis adjusted for possible confounding.

**Results:**

A total of 150,949 patients were included. The mean age of the cohort was 74 years (48.2% female). The mean follow-up time was 3.3 years. The combined effectiveness endpoint of ischaemic events (transient ischaemic attack, systemic embolism, pulmonary embolism, or ischaemic stroke) showed the following results compared to acenocoumarol: warfarin (RR:1.06; 95%CI 0.93–1.22); dabigatran (RR:1.17; 95%CI 1.02–1.33); rivaroxaban (RR:1.15; 95%CI 1.05–1.26); apixaban (RR: 0.96; 95%CI 0.87–1.07) and edoxaban (RR: 1.10; 95%CI 0.79–1.51). Compared to acenocoumarol, the risk of all-cause mortality was lower for dabigatran, rivaroxaban and apixaban (RR:0.77; 95%CI 0.72–0.82; RR:0.79; 95%CI 0.76–0.83; RR:0.85; 95%CI 0.81–0.89, respectively) and higher for warfarin (RR:1.12; 95%CI 1.05–1.20). An increased risk of gastrointestinal bleeding was observed with dabigatran (RR:1.36; 95%CI 1.09–1.70) and a lower risk with rivaroxaban (RR:0.84; 95%CI 0.72–0.98). All 4 DOACs showed a lower risk of intracranial bleeding compared to acenocoumarol. Warfarin carried a higher risk of both gastrointestinal bleeding (RR:1.64; 95%CI 1.31–2.06) and intracranial bleeding (RR:1.61; 95%CI 1.22–2.13) compared to acenocoumarol. An unadjusted analysis of matched groups in a multivariate Cox regression analysis yielded similar results for combined effectiveness and safety outcomes compared to acenocoumarol.

**Conclusion:**

Although DOACs were clearly associated with a lower risk of intracranial bleeding compared to acenocoumarol, our data did not reveal a significant reduction in thromboembolic events. Warfarin was found to be both less effective and less safe than acenocoumarol.

## Introduction

Preventing ischaemic or thromboembolic events is one of the primary goals of anticoagulation therapy for patients with atrial fibrillation (AF). Before direct-acting oral anticoagulants (DOACs) became available, vitamin K antagonists (VKAs) such as warfarin or acenocoumarol were the preferred first-line agents for preventing ischaemic events in patients with AF (January et al., 2019; Hindricks et al., 2021).

Clinical trials investigating the use of DOACs in the treatment of AF were conducted with warfarin as the comparator. These trials showed that DOACs had similar efficacy but posed a lower risk of intracranial haemorrhage when compared with warfarin (Connolly et al., 2009; Granger et al., 2011; Patel et al., 2011; Giugliano et al., 2013). In light of the results, and given the fact that DOACs offer simpler dosing, fewer drug and food interactions and do not require anticoagulation monitoring (of the International Normalized Ratio, INR), some scientific societies and clinical practice guidelines now recommend DOACs as the first-line agents in patients with AF, replacing the use of vitamin K antagonists (January et al., 2019; Hindricks et al., 2021).

We decided to conduct this study for several reasons. Firstly, no clinical trials have been identified that compare different DOACs head-to-head. Meanwhile, observational studies have found differences in the outcomes of each DOAC compared to warfarin, as well as in the outcomes of clinical trials (Nielsen et al., 2017; Tepper et al., 2018; Chan, Lee, Chao, et al., 2019; Chan, Lee, See, et al., 2019; Ramagopalan et al., 2019; Xue and Zhang, 2019; Douros et al., 2019; Xue et al., 2020; Domek et al., 2020; Lip et al., 2021; Durand et al., 2021; Ingason et al., 2021; Kim et al., 2021; Li et al., 2021; Mamas et al., 2022; Jaksa et al., 2022; Lau et al., 2022).

Furthermore, while warfarin is the reference VKA drug in the majority of studies conducted in AF patients, acenocoumarol is the drug of choice in certain populations, such as Spain and the Netherlands (Rosa et al., 2018; Rodríguez-Bernal et al., 2021). One possible reason why the results obtained with warfarin in clinical trials and observational studies are not generalisable to acenocoumarol is the difference in their half-lives. When extrapolating data to real-world practice, compliance should also be considered. Research findings from one observational study have indicated that patients with AF who were prescribed DOACs were three times more likely to be non-adherent than those prescribed VKAs (Rodriguez-Bernal et al., 2018). Adherence issues, as well as other contributing factors, such as comorbidities, concomitant medications, age or sex, may affect the effectiveness of oral anticoagulants used in actual clinical practice. Finally, no clinical trials or observational studies have compared the efficacy, effectiveness or safety of edoxaban, which is the most recently marketed DOAC, with acenocoumarol.

The main aim of this study was to evaluate the effectiveness (ischaemic/transient stroke, systemic/pulmonary embolism and mortality) and safety (gastrointestinal and intracranial bleeding) of DOACs (dabigatran, rivaroxaban, apixaban and edoxaban) and warfarin versus acenocoumarol in a large population cohort under real-world clinical practice conditions with 8 years of follow-up.

## Materials and methods

### Study design

This retrospective, real-world, data-based study of patients with AF aimed to analyse the effectiveness and safety of oral anticoagulant (OAC) therapy (warfarin, dabigatran, rivaroxaban, apixaban and edoxaban) compared to acenocoumarol (Montero-Balosa et al., 2023). In an attempt to simulate a randomised experiment (quasi-experimental study), all outcomes were analysed using intention-to-treat (ITT) and propensity score matching. The study period ran from January 2012 to December 2020.

### Population and setting

The study was carried out in the Autonomous Community of Andalusia (Spain). The Andalusian Health Service (AHS) provides approximately 8.5 million people in this region with free universal healthcare at the point of use.

Patients in the study were seen in consultations at any level of healthcare (primary care or hospital) within the AHS and met the following inclusion criteria: they were over 40 years of age, had received a diagnosis of atrial fibrillation (AF) based on ICD-9 or ICD-10 (International Classification of Diseases) codes and were initiating treatment with oral anticoagulants (OACs) in the context of routine clinical practice (naïve population). Only new OAC users were included. This meant those who had previously taken OACs were included as long as provided that they had stopped taking them in the 12 months prior to their inclusion in the study.

Patients were excluded if they had severe mitral stenosis, a diagnosis of valvular heart disease, or were undergoing aortic and/or mitral valve procedures, as defined by the ICD-9 or ICD-10 codes.

A more detailed description of the ICD-9 and ICD-10 codes can be found in the published SIESTA-A study protocol (Montero-Balosa et al., 2023).

### Data sources

The data source for this study was Andalusia’s Population Health Database (BPS, Base Poblacional de Salud, Spanish acronym). This population-based health information system collects clinical data of every individual receiving healthcare in the AHS, as well as information on their use of health resources (Muñoyerro-Muñiz et al., 2020). This information is stored in different databases, which are linked by each patient’s unique identification number in the AHS. The information contained in this population health database includes patient affiliations, socio-demographics, diagnosed health problems, cardiovascular risk factors and lifestyles, utilisation of resources in outpatient consultations, primary care, hospital care and emergency care, hospital admissions, mortality, and prescription and pharmacy dispensing data in primary and hospital care (Montero-Balosa et al., 2023).

### Outcomes

The effectiveness outcomes (dependent variables) were defined as the incidence of a new first event from the following list: transient ischaemic attack (TIA), systemic embolism, pulmonary embolism, ischaemic stroke, or all-cause mortality. These were considered a combined effectiveness endpoint (Montero-Balosa et al., 2023).

The safety outcomes were defined as the incidence of a new first event of major bleeding leading to hospital admission (gastrointestinal or intracranial bleeding). These were considered a combined safety endpoint (Montero-Balosa et al., 2023). Post-traumatic bleeding was excluded.

All effectiveness and safety events were associated with the date of onset and identified by the relevant ICD-9 or ICD-10 code when the patient was treated in hospital. Diagnoses of TIA with a recorded date were collected from both hospitals and primary care centres.

The index date (i.e., the date of the first treatment) was defined as the date on which a prescribed anticoagulant treatment was dispensed from the pharmacy, provided that no other anticoagulant had been prescribed in the previous 12 months.

Follow-up time was defined as the number of days between the index date of treatment initiation (i.e., pharmacy dispensing) and the date of the first new diagnosis, death or the end of the study period (31 December 2020).

### Independent variables

The independent variables included in the multivariate survival analysis were the type of OAC therapy, sociodemographic data (age, sex, place of residence, approximate income based on pharmaceutical co-payment status), comorbidities, medication use, CHADS-VASC (thromboembolic risk assessment) and HAS-BLED (bleeding risk assessment) scores. All of these were assessed at study entry. Frequency of contact with the AHS (primary care, hospital, home visits, and emergency department) was also considered (Montero-Balosa et al., 2023).

Comorbidities were defined as an active diagnosis (ICD-9 or ICD-10) recorded in one of the population health databases (emergency, hospital or primary care) in the 12 months prior to the date of treatment initiation. Medications that increase or reduce bleeding risk were included in the HAS-BLED calculation (Montero-Balosa et al., 2023). All predictor variables were analysed for their association with events, based on the information available in the 12 months prior to the index date.

### Statistical analysis

Descriptive statistics were calculated and all outcomes were analysed using the intention-to-treat (ITT) method. Patients were categorised according to the initial OAC treatment started during the study period, provided that they had not taken any other OAC for at least 12 months prior to the study (“washout period”) and regardless of possible changes in OAC therapy during the follow-up period.

When measuring the effectiveness and safety outcomes, only the first recorded instance of each type of event included in the study was considered. If a primary outcome variable was identified in two or more study databases, the first recorded date was used.

Quantitative variables were summarised using means and standard deviation. Non-quantitative variables were summarised using frequency tables and percentages. Point estimates and 95% confidence intervals were obtained for the various statistical analyses. Incidence rates were calculated by dividing the number of events by the corresponding person-time of follow-up.

### Sensitivity analysis

In addition to the main analysis, sensitivity analyses were performed to consider the effect of sex and the exclusion of mortality from the combined effectiveness variable. The TIAs were also studied, selecting only those with a confirmed hospital diagnosis. The Fine-Gray model was used to analyse the competing risk of death (Austin and Fine, 2017).

### Phases of the analysis

The analysis comprised three phases. The first phase consisted of a descriptive analysis of the total number of individuals included in the study, and of the patients in each of the 6 cohorts, which were grouped according to the type of initial anticoagulant taken. Next, the incidence of effectiveness and safety events over the 8 years of follow-up was analysed.

In the second phase, Propensity Score Matching (PSM) was performed. The nearest neighbor matching approach was used to compare patients taking the 4 DOACs or warfarin to patients taking acenocoumarol (the reference drug).

Propensity Score (PS) values (between 0 and 1) indicated the a priori probability that a patient would receive treatment with acenocoumarol compared to the OAC under comparison. PS scores were calculated using logistic regression models to predict the binary dependent variable. Initially, the following independent variables were included: age, sex, province, pharmaceutical co-payment status, use of healthcare resources by the patient, comorbidities, use of concomitant medications at study entry associated with an increased risk of bleeding (acetylsalicylic acid, other antiplatelet agents, heparins, glucocorticoids, non-steroidal anti-inflammatory drugs, selective serotonin reuptake inhibitors, macrolides) as well as those associated with a reduced risk of bleeding (proton pump inhibitors, H2-receptor antagonists, antihaemorrhagic drugs) and the CHADS-VASC and HAS-BLED scores (Montero-Balosa et al., 2023).

Mean differences for quantitative variables and differences of distribution for categorical variables after PSM were then compared with the differences observed in the original 6 cohorts. The “matchit” function in the Matchit package (R version 4.1.0) was used for the matching analysis to perform pairing, subset selection and subclassification to ensure covariate balance between treatment and control groups.

After matching, the incidence rate of events (person-time in follow-up) was analysed in relation to the effectiveness and safety of the treatment over the 8-year follow-up period. Only the cases selected for each pair of oral anticoagulants were compared (acenocoumarol was used as the reference). Bivariate survival analysis (for time-censored data) was performed using Kaplan-Meier curves to plot the frequency of different events or diseases occurring over time. Survival probabilities were calculated by generating statistics and plotting survival functions for each study group. The log-rank test was then used to test for equality of survival time distributions between the different groups.

Multivariate survival analysis was performed on the combined effectiveness and safety outcomes. Cox regression was used to create time-to-event models using both categorical and continuous predictor variables (covariates) as hypothesised predictors. In addition to Cox regression, a Fine-Gray analysis was conducted to consider the competing risk of death. First, we tested whether the OAC versus acenocoumarol survival curves crossed in the matched groups and whether the proportional hazards assumption was met visually over time in the Kaplan-Meier curves. The risk of outcomes (effectiveness and safety) in DOAC or warfarin users versus acenocoumarol users (reference) was determined by calculating specific instantaneous risk ratios (hazard ratio, HR) for each comparison using multivariate Cox regression models.

A matched-pair analysis was performed for the period 2013–2020. For the acenocoumarol-apixaban and acenocoumarol-edoxaban pairs, which were launched after 2013, the analysis periods were 2014–2020 and 2017–2020, respectively.

The information was extracted from the population health databases and transferred into a single file. This file was then analysed using R (version 4.1.0), and Stata (version 14 for Windows) statistical software. Patients were anonymised for evaluation by the study investigators.

The study was reported in accordance with the Strengthening the Reporting of Observational Studies in Epidemiology (STROBE) statement for cohort studies (Gharaibeh et al., 2014).

### Registration details

The study protocol was submitted to the Spanish Agency of Medicines and Medical Devices (AEMPS, Spanish acronym) and classified as an observational study (AEMPS reference number: 0004–2022-OBS; 12 January 2022).

## Results

**FIGURE 1:**
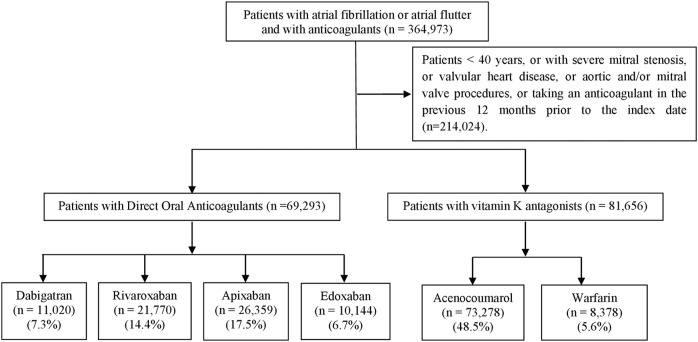
Flowchart for cohort selection.

After applying the inclusion and exclusion criteria, a total of 150,949 patients were included in the six study cohorts (Figure 1).

The most common comorbidities were hypertension (77.7%), diabetes (35.8%), cardiac arrhythmias (25.4%), heart failure (21.6%) and chronic obstructive pulmonary disease (COPD, 23.7%). The mean age of the population was 74 years (SD:10.2), and 48.2% of patients were female (n = 72,817) (Table 1).

**TABLE 1:** Cohort characteristics at baseline by type of anticoagulant.

Characteristics	Total (N = 150,949)	Acenocoumarol (N = 73,278)	Warfarin (N = 8,378)	Dabigatran (N = 11,020)	Rivaroxaban (N = 21,770)	Apixaban (N = 26,359)	Edoxaban (N = 10,144)
Age (years) n (%)							
40–59	13,767 (9.1)	5,524 (7.5)	759 (9.1)	1,477 (13.4)	2,509 (11.5)	2,309 (8.8)	1,189 (11.7)
60–69	30,926 (20.5)	13,952 (19)	1,814 (21.7)	2,822 (25.6)	4,989 (22.9)	5,060 (19.2)	2,289 (22.6)
70–79	5,3850 (35.7)	27,115 (37)	2,980 (35.6)	3,672 (33.3)	7,738 (35.5)	8,841 (33.5)	3,504 (34.5)
80–89	46,884 (31.1)	24,320 (33.2)	2,540 (30.3)	2,756 (25)	5,852 (26.9)	8,675 (32.9)	2,741 (27)
90–112	5,522 (3.7)	2,367 (3.2)	285 (3.4)	293 (2.7)	682 (3.1)	1,474 (5.6)	421 (4.2)
mean (SD)	74 (10.2)	75 (9.7)	74 (10.1)	72 (10.8)	73 (10.5)	75 (10.5)	73 (10.8)
Sex N (%)							
MALE	78,120 (51.8)	37,146 (50.7)	4,309 (51.4)	6,100 (55.4)	11,728 (53.9)	13,441 (51)	5,396 (53.2)
FEMALE	72,817 (48.2)	36,129 (49.3)	4,068 (48.6)	4,919 (44.6)	10,040 (46.1)	12,915 (49)	4,746 (46.8)
Baseline risk of stroke (chads-vasc) and bleeding (has-bled), mean (sd)							
CHADS-VASC score	3.5 (1.7)	3.6 (1.6)	3.6 (1.7)	3.2 (1.7)	3.3 (1.7)	3.6 (1.7)	3.3 (1.7)
HAS-BLED score	2.4 (1)	2.5 (1)	2.4 (1)	2.2 (1.1)	2.3 (1)	2.4 (1)	2.3 (1.1)
Comorbidities, n (%)							
Prior peptic ulcer	3,192 (2.1)	1,472 (2)	165 (2)	218 (2)	437 (2)	632 (2.4)	268 (2.6)
Cancer	26,502 (17.6)	12,630 (17.2)	1,458 (17.4)	1,782 (16.2)	3,513 (16.1)	5,132 (19.5)	1,987 (19.6)
Thyroid disease	20,973 (13.9)	9,983 (13.6)	1,255 (15)	1,409 (12.8)	3,088 (14.2)	3,721 (14.1)	1,517 (15)
Diabetes mellitus	54,044 (35.8)	27,037 (36.9)	3,191 (38.1)	3,595 (32.6)	7,282 (33.4)	9,604 (36.4)	3,335 (32.9)
Dementia	6,887 (4.6)	3,405 (4.6)	345 (4.1)	403 (3.7)	937 (4.3)	1,373 (5.2)	424 (4.2)
Sleep apnea	10,910 (7.2)	4,995 (6.8)	723 (8.6)	811 (7.4)	1,633 (7.5)	1,897 (7.2)	851 (8.4)
Hypertension	11,7264 (77.7)	57,543 (78.5)	6,522 (77.8)	8,236 (74.7)	16,465 (75.6)	20,806 (78.9)	7,692 (75.8)
Myocardial infarction	10,577 (7)	5,036 (6.9)	766 (9.1)	732 (6.6)	1,459 (6.7)	1,948 (7.4)	636 (6.3)
Angina pectoris	18,594 (12.3)	8,958 (12.2)	1,276 (15.2)	1,235 (11.2)	2,635 (12.1)	3,313 (12.6)	1,177 (11.6)
Cardiac arrhytmias	38,381 (25.4)	18,471 (25.2)	2,740 (32.7)	2,807 (25.5)	6,163 (28.3)	6,071 (23)	2,129 (21)
Heart failure	32,534 (21.6)	16,894 (23.1)	2,120 (25.3)	1,859 (16.9)	4,482 (20.6)	5,458 (20.7)	1,721 (17)
Prior stroke	6,595 (4.4)	3,082 (4.2)	381 (4.5)	450 (4.1)	855 (3.9)	1,442 (5.5)	385 (3.8)
Peripheral vascular disease	11,352 (7.5)	5,646 (7.7)	759 (9.1)	701 (6.4)	1,484 (6.8)	2,073 (7.9)	689 (6.8)
Venous thrombosis	12,042 (8)	6,257 (8.5)	673 (8)	670 (6.1)	1,586 (7.3)	2,106 (8)	750 (7.4)
Copd	35,722 (23.7)	18,259 (24.9)	2,003 (23.9)	2,338 (21.2)	4,693 (21.6)	6,137 (23.3)	2,292 (22.6)
Liver disease	1,031 (0.7)	509 (0.7)	81 (1)	66 (0.6)	125 (0.6)	194 (0.7)	56 (0.6)
Chronic renal failure	12,129 (8)	6,832 (9.3)	657 (7.8)	430 (3.9)	1,339 (6.2)	2,220 (8.4)	651 (6.4)
Events at study entry, n (%)							
Transient ischaemic attack	8,695 (5.8)	4,067 (5.6)	468 (5.6)	669 (6.1)	1,152 (5.3)	1,815 (6.9)	524 (5.2)
Systemic embolism	137 (0.1)	61 (0.1)	13 (0.2)	8 (0.1)	16 (0.1)	29 (0.1)	10 (0.1)
Pulmonary embolism	303 (0.2)	190 (0.3)	21 (0.3)	11 (0.1)	27 (0.1)	40 (0.2)	14 (0.1)
Ischaemic stroke	509 (0.3)	177 (0.2)	30 (0.4)	61 (0.6)	47 (0.2)	152 (0.6)	42 (0.4)
Gastrointestinal bleeding	6,127 (4.1)	2,865 (3.9	389 (4.6)	383 (3.5)	775 (3.6)	1,309 (5)	406 (4)
Intracranial bleeding	1,994 (1.3)	684 (0.9)	72 (0.9)	234 (2.1)	307 (1.4)	551 (2.1)	146 (1.4)

COPD, chronic obstructive pulmonary disease.

The mean follow-up time was 3.3 years: 3.8 years for acenocoumarol, 3.6 years for warfarin, 3.7 years for dabigatran, 3.3 years for rivaroxaban, 2.5 years for apixaban and 1.6 years for edoxaban.

At study entry, patients treated with dabigatran were younger (with a higher proportion in the 40–59 age group). They also had the lowest mean CHADS-VASC and HAS-BLED scores, as well as a lower prevalence of hypertension, renal insufficiency, diabetes and heart failure. Patients on apixaban had a higher prevalence of previous stroke, dementia, previous gastrointestinal bleeding and previous intracranial bleeding. Those on edoxaban showed a lower prevalence of myocardial infarction, arrhythmia and previous stroke. Patients on warfarin had a higher prevalence of diabetes, angina, myocardial infarction, arrhythmia, heart failure and peripheral arterial disease. Finally, patients treated with acenocoumarol had a higher prevalence of venous thrombosis, COPD and renal failure compared to those treated with the other five anticoagulants (Table 1).

At the beginning of the study, patients taking acenocoumarol or warfarin were more likely to be taking heparins and antithrombotics (18.6% and 17.7%, respectively) than those taking DOACs (range: 4.8%–8.8%) and were also more likely to be taking antiplatelet agents (27.1% and 31.6%, respectively) than those taking DOACs (range: 21.9%–26.6%). Patients receiving warfarin treatment had a higher consumption of H2 blockers and gastroprotective drugs (9.3%) than those on DOACs (range: 5.0%–6.6%) (Table 2).

**TABLE 2:** Utilisation of medication and healthcare resources during follow-up by province, pharmaceutical co-payment status and anticoagulant.

Characteristics	TOTAL (N = 150,949)	ACENOCOUMAROL (N = 73,278)	WARFARIN (N = 8,378)	DABIGATRAN (N = 11,020)	RIVAROXABAN (N = 21,770)	APIXABAN (N = 26,359)	EDOXABAN (N = 10,144)
Medication, n (%)							
Heparins and antithrombotics	19,276 (12.8)	13,657 (18.6)	1,487 (17.7)	531 (4.8)	1,140 (5.2)	1,566 (5.9)	895 (8.8)
Antiplatelets	39,448 (26.1)	19,827 (27.1)	2,646 (31.6)	2,869 (26)	4,771 (21.9)	7,004 (26.6)	2,331 (23)
Systemic corticosteroids	15,637 (10.4)	8,152 (11.1)	604 (7.2)	923 (8.4)	2,131 (9.8)	2,861 (10.9)	966 (9.5)
Nsad	44,767 (29.7)	22,433 (30.6)	2,333 (27.8)	3,090 (28)	6,447 (29.6)	7,458 (28.3)	3,006 (29.6)
S. serotonin reuptake inhib.	17,042 (11.3)	8,113 (11.1)	881 (10.5)	1,246 (11.3)	2,337 (10.7)	3,263 (12.4)	1,202 (11.8)
Macrolide antibiotics	6,489 (4.3)	3,027 (4.1)	275 (3.3)	415 (3.8)	1,043 (4.8)	1,237 (4.7)	492 (4.9)
H2 Blockers/gastro-protect.	9,107 (6)	4,276 (5.8)	777 (9.3)	725 (6.6)	1,232 (5.7)	1,594 (6)	503 (5)
Proton pump inhibitors	95,630 (63.4)	46,589 (63.6)	4,707 (56.2)	7,200 (65.3)	13,575 (62.4)	17,493 (66.4)	6,066 (59.8)
Antihemorragic agents	891 (0.6)	555 (0.8)	67 (0.8)	46 (0.4)	72 (0.3)	107 (0.4)	44 (0.4)
Pharmaceutical co-payment status, n (%)							
Employed	11,548 (7.7)	3,895 (5.3)	558 (6.7)	1,217 (11)	2,348 (10.8)	2,239 (8.5)	1,291 (12.7)
Pensioners	92,859 (61.5)	46,271 (63.1)	5,506 (65.7)	6,572 (59.6)	13,237 (60.8)	15,775 (59.8)	5,498 (54.2)
Exempt from payment	45,673 (30.3)	22,649 (30.9)	2,288 (27.3)	3,163 (28.7)	6,025 (27.7)	8,211 (31.2)	3,337 (32.9)
Others and not specified	869 (0.6)	463 (0.6)	26 (0.3)	68 (0.6)	160 (0.7)	134 (0.5)	18 (0.2)
Province, n (%)							
Almería	10,443 (6.9)	5,420 (7.4)	48 (0.6)	1,021 (9.3)	2,326 (10.7)	1,224 (4.6)	404 (4)
Cádiz	20,322 (13.5)	8,623 (11.8)	97 (1.2)	1,707 (15.5)	3,569 (16.4)	4,645 (17.6)	1,681 (16.6)
Córdoba	16,485 (10.9)	7,753 (10.6)	55 (0.7)	1,184 (10.7)	2,216 (10.2)	3,423 (13)	1,854 (18.3)
Granada	16,290 (10.8)	13,083 (17.9)	173 (2.1)	431 (3.9)	1,067 (4.9)	1,263 (4.8)	273 (2.7)
Huelva	9,552 (6.3)	2,970 (4.1)	73 (0.9)	680 (6.2)	1,865 (8.6)	2,762 (10.5)	1,202 (11.8)
Jaén	13,493 (8.9)	4,901 (6.7)	31 (0.4)	1,550 (14.1)	2,554 (11.7)	3,579 (13.6)	878 (8.7)
Málaga	26,527 (17.6)	9,136 (12.5)	199 (2.4)	2,655 (24.1)	5,353 (24.6)	6,862 (26)	2,322 (22.9)
Sevilla	32,969 (21.8)	18,265 (24.9)	7,490 (89.4)	1,492 (13.5)	2,264 (10.4)	2,056 (7.8)	1,402 (13.8)
Not specified	4,868 (3.2)	3,127 (4.3)	212 (2.5)	300 (2.7)	556 (2.6)	545 (2.1)	128 (1.3)
Resource use, mean (SD)							
Outpatient consultations	414.2 (308.2)	425.8 (322.1)	508.1 (368)	397 (285.5)	390 (285.2)	390.6 (279.3)	385.6 (272.9)
Primary care emergency	108.3 (187.9)	111.5 (192.6)	151.2 (241.6)	99.2 (189.9)	101.1 (176.6)	100.9 (169.1)	93.8 (163.3)
Hospital emergency	103.1 (117.9)	112.5 (123.1)	101.9 (102.6)	92.8 (106.1)	95 (132.3)	95.3 (103)	85.2 (99.3)
Primary care consultations	1,905.9 (1197.7)	2,222.1 (1,270.6)	2,153.6 (1,198.7)	1,542.8 (1,050.6)	1,524.7 (997.2)	1,581.1 (997.3)	1,473.8 (943.9)
Home visits	371.5 (776.7)	490.6 (894)	513 (931.7)	227.4 (615.2)	236.5 (592.7)	250 (580.9)	155.8 (424.7)
Hospital admission	28.2 (40.7)	30.4 (44.3)	35.2 (48)	25.9 (38.8)	24.9 (37)	26.8 (34.5)	19.5 (27.1)

NSAID, Non-steroidal anti-inflammatory drug; Resource Use: mean number of consultations or admissions/100 person-years of follow-up.

In terms of socioeconomic status, treatment with a DOAC was more common among patients who contributed 40%–50% of the drug’s cost (i.e., younger, employed patients). In contrast, retired patients who contributed less than 10% of the cost were more likely to be treated with a VKA (Table 2).

During the follow-up period, utilisation of health services (outpatient appointments, home visits and primary care emergencies) was higher among patients taking acenocoumarol or warfarin than among those treated with DOACs (Table 2).

After PSM, the differences in means and categorical distributions of the various variables were examined and compared with those observed in the original six cohorts. The distances between variables were reduced in the matched groups, indicating greater similarity between them, thereby allowing for a more accurate comparison of the treatment effect and increasing the validity of the results (Supplementary Table S1). The selection of probability ranges and overlaps for matching patients undergoing treatment with each pair of anticoagulants is shown in Supplementary Figure S1.

### Effectiveness results

The first analysis of incidence rates, using number of events per 1,000 person-years of follow-up within matched groups (PSM), showed that dabigatran (RR:0.84; 95%CI 0.79–0.89), rivaroxaban (RR:0.85; 95%CI 0.82–0.88), and apixaban (RR:0.88; 95%CI 0.84–0.91) were more effective on the combined effectiveness endpoint compared to acenocoumarol, while warfarin was less effective (RR:1.11; 95%CI 1.05–1.18) (Table 3).

**TABLE 3:** Matched group comparisons of each anticoagulant versus acenocoumarol (propensity score matching): Relative risk and 95% confidence intervals for combined effectiveness, combined safety, and specific event types.

Type of event during follow-up	Warfarin (n = 14,942)	Dabigatran (n = 19,424)	Rivaroxaban (n = 41,748)	Apixaban^a^ (n = 36,862)	Edoxaban^a^ (n = 6,160)
Effectiveness					
Combined Effectiveness^b^	1.11 (1.05–1.18)	0.84 (0.79–0.89)	0.85 (0.82–0.88)	0.88 (0.84–0.91)	0.95 (0.82–1.09)
Transient ischaemic attack	0.90 (0.73–1.09)	1.15 (0.95–1.39)	1.18 (1.04–1.34)	1.17 (1.01–1.35)	0.87 (0.52–1.42)
Systemic embolism	2.05 (1.16–3.76)	1.25 (0.66–2.37)	0.84 (0.54–1.28)	0.84 (0.51–1.35)	3.38 (0.73–23.60)
Pulmonary embolism	1.08 (0.59–1.97)	0.62 (0.34–1.07)	0.76 (0.54–1.08)	1.18 (0.84–1.65)	0.49 (0.14–1.44)
Ischaemic stroke	1.18 (1.00–1.39)	1.11 (0.94–1.31)	1.08 (0.97–1.21)	0.86 (0.75–0.98)	1.14 (0.76–1.71)
All-cause mortality	1.12 (1.05–1.20)	0.77 (0.72–0.82)	0.79 (0.76–0.83)	0.85 (0.81–0.89)	0.92 (0.78–1.09)
Safety					
Combined Safety^c^	1.63 (1.37–1.95)	0.88 (0.74–1.04)	0.73 (0.64–0.82)	0.85 (0.74–0.97)	0.71 (0.44–1.13)
Gastrointestinal bleeding	1.64 (1.31–2.06)	1.36 (1.09–1.70)	0.84 (0.72–0.98)	0.87 (0.73–1.04)	1.18 (0.64–2.15)
Intracranial bleeding	1.61 (1.22–2.13)	0.42 (0.31–0.77)	0.58 (0.47–0.71)	0.81 (0.65–0.99)	0.31 (0.11–0.72)

^a^ Apixaban and edoxaban are analysed from the year they were marketed; apixaban: 2014–2020; edoxaban: 2017–2020.

^b^ Combined effectiveness: transient ischaemic attack, systemic embolism, pulmonary embolism, ischaemic stroke or all-cause mortality.

^c^ Combined safety, gastrointestinal or intracranial bleeding.

Following the second analysis, which excluded all-cause mortality from the combined effectiveness outcome, dabigatran and rivaroxaban were found to be associated with an increased risk of ischaemic events (RR:1.17; 95%CI 1.02–1.33; RR:1.15; 95%CI 1.05–1.26, respectively) (Table 4). After sensitivity analysis (using the Fine-Gray model) to account for the competing risk of death, the results were consistent for all the anticoagulants under study (Table 5).

**TABLE 4:** Sensitivity analysis of matched groups of anticoagulants versus acenocoumarol (propensity score matching). Relative risk and 95% confidence intervals for all ischaemic events and transient ischaemic attack for the total population and according to sex.

(A) Total Population					
Type of event during follow-up	Warfarin (n = 14,942)	Dabigatran (n = 19,424)	Rivaroxaban (n = 41,748)	Apixaban ^a^ (n = 36,862)	Edoxaban ^a^ (n = 6,160)
Ischaemic events ^b^	1.06 (0.93–1.22)	1.17 (1.02–1.33)	1.15 (1.05–1.26)	0.96 (0.87–1.07)	1.10 (0.79–1.51)
Transient ischaemic attack^c^	0.79 (0.59–1.06)	1.24 (0.96–1.62)	1.20 (1.01–1.43)	1.22 (1.01–1.47)	1.01 (0.52–1.93)

(B) Females					
Type of event during follow-up	Warfarin (n = 3,490)	Dabigatran (n = 3,912)	Rivaroxaban (n = 9,668)	Apixaban ^a^ (n = 8,806)	Edoxaban ^a^ (n = 2,318)
Ischaemic events ^b^	1.18 (0.89–1.56)	1.40 (1.07–1.84)	1.25 (1.05–1.49)	0.94 (0.76–1.16)	1.06 (0.63–1.79)
Transient ischaemic attack3	0.80 (0.44–1.47)	1.18 (0.69–2.01)	1.17 (0.85–1.62)	1.21 (0.81–1.81)	0.50 (0.11–1.72)

(C) Males					
Type of event during follow-up	Warfarin (n = 4,494)	Dabigatran (n = 6,668)	Rivaroxaban (n = 12,516)	Apixaban ^a^ (n = 9,930)	Edoxaban ^a^ (n = 2,846)
Ischaemic events^b^	1.00 (0.78–1.29)	1.18 (0.94–1.49)	1.06 (0.89–1.26)	1.08 (0.89–1.32)	1.02 (0.63–1.65)
Transient ischaemic attack^c^	0.90 (0.52–1.55)	1.01 (0.65–1.57)	1.35 (0.96–1.89)	1.60 (1.10–2.35)	1.51 (0.58–4.03)

^a^ Apixaban and edoxaban are analysed from the year they were marketed; apixaban: 2014–2020; edoxaban: 2017–2020.

^b^ Ischaemic events: transient ischaemic attack, systemic embolism, pulmonary embolism or ischaemic stroke.

^c^ Transient ischaemic attack: only those confirmed hospital diagnosis.

**TABLE 5:** Sensitivity analysis of matched group of anticoagulants versus acenocoumarol (propensity score matching): Results of the Fine-Gray model for competing risk of death, with subdistribution hazard ratios and 95% confidence intervals for ischaemic events and combined safety events.

Type of event during follow-up	Warfarin (n = 14,942)	Dabigatran (n = 19,424)	Rivaroxaban (n = 41,748)	Apixaban ^a^ (n = 36,862)	Edoxaban ^a^ (n = 6,160)
Effectiveness					
Ischaemic events^b^	1.09 (0.95–1.26)	1.11 (0.97–1.27)	1.10 (1.00–1.20)	0.87 (0.78–0.96)	0.94 (0.68–1.30)
Safety					
Combined Safety^c^	1.61 (1.35–1.91)	0.88 (0.74–1.04)	0.73 (0.65–0.82)	0.83 (0.73–0.95)	0.61 (0.39–0.94)

^a^ Apixaban and edoxaban are analysed from the year they were marketed; apixaban: 2014–2020; edoxaban: 2017–2020.

^b^ Ischaemic events: transient ischaemic attack, systemic embolism, pulmonary embolism or ischaemic stroke.

^c^ Combined safety: gastrointestinal or intracranial bleeding.

An analysis of specfic event types in this study revealed an increased risk of TIA with rivaroxaban (RR: 1.18; 95%CI 1.04–1.34) and apixaban (RR: 1.17; 95%CI 1.01–1.35), and a lower risk of ischaemic stroke with apixaban (RR: 0.86; 95%CI 0.75–0.98) compared to acenocoumarol. Warfarin was less effective than acenocoumarol in preventing systemic embolism (RR: 2.05; 95%CI 1.16–3.76) (Table 3).

**FIGURE 2:**
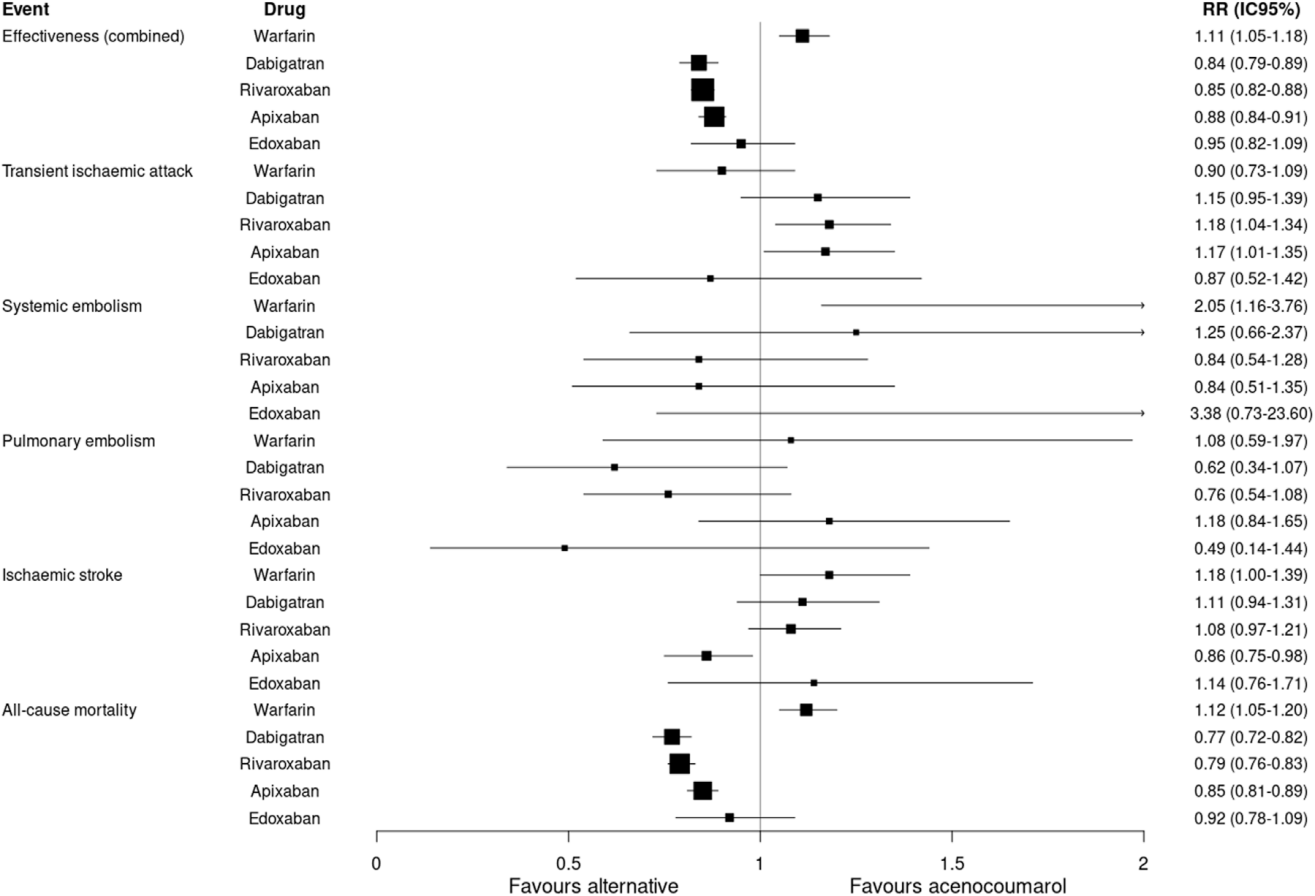
Effectiveness outcomes. Relative risk and 95% confidence intervals for combined effectiveness and specific event types. Effectiveness (combined): transient ischaemic attack, systemic embolism, pulmonary embolism, ischaemic stroke or all-cause mortality.

The risk of death was lower for dabigatran, rivaroxaban and apixaban (RR: 0.77; 95%CI 0.72–0.82; RR: 0.79; 95%CI 0.76–0.83; RR: 0.85; 95%CI 0.81–0.89, respectively) and higher for warfarin compared to acenocoumarol (RR: 1.12; 95%CI 1.05–1.20) (Table 3; Figure 2).

### Safety results

**FIGURE 3:**
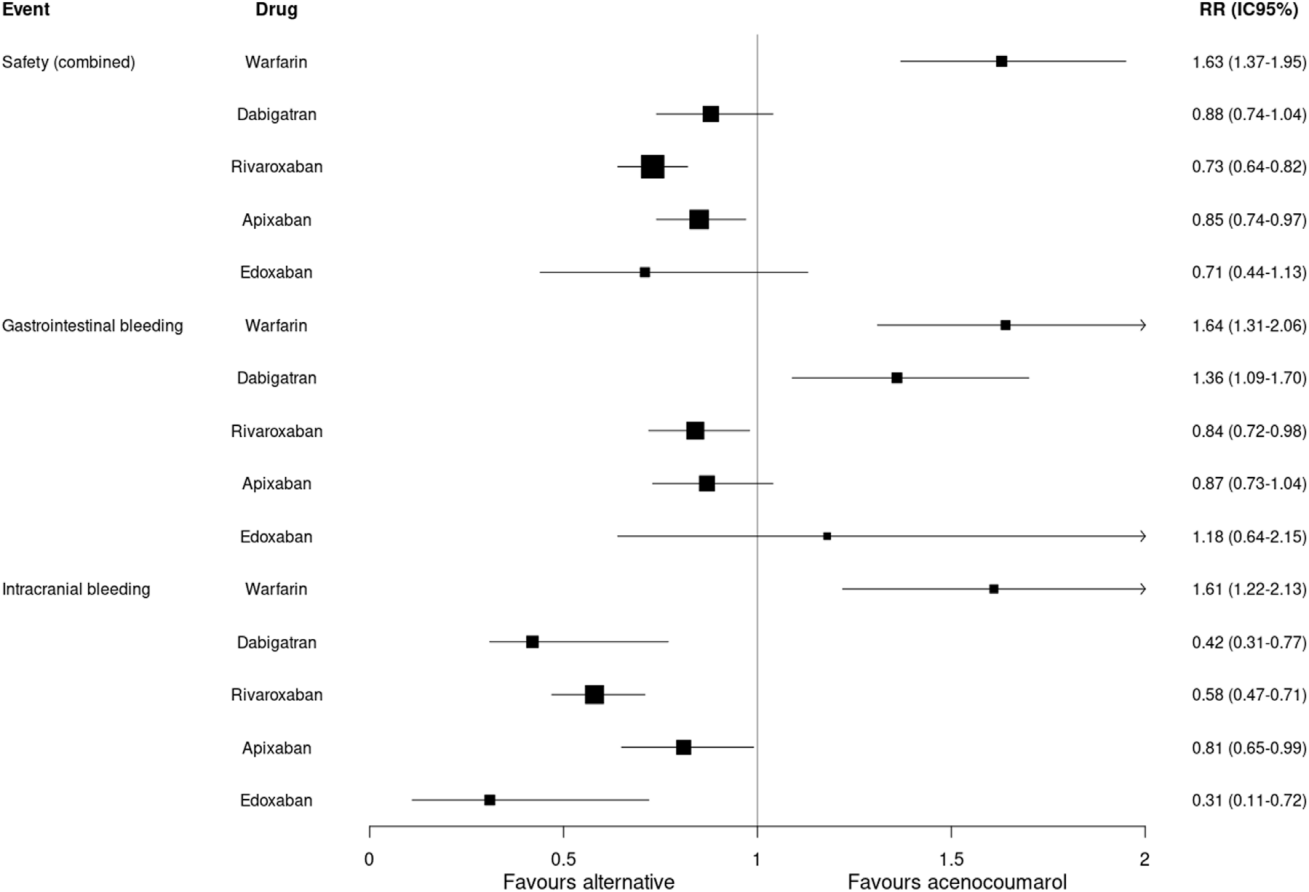
Safety outcomes. Relative risk and 95% confidence intervals for combined safety and specific event types. Safety (combined): gastrointestinal or intracranial bleeding.

The analysis of incidence rates by matched cohorts (PSM) showed a lower incidence of combined safety events with rivaroxaban (RR: 0.73; 95%CI 0.64–0.82) and apixaban (RR: 0.85; 95%CI 0.74–0.97) compared to acenocoumarol. An increased risk of gastrointestinal bleeding was observed with dabigatran (RR: 1.36; 95%CI 1.09–1.70) and a lower risk with rivaroxaban (RR: 0.84; 95%CI 0.72–0.98). The risk of intracranial bleeding was lower for all 4 DOACs compared to acenocoumarol. Warfarin had a higher risk of combined safety events (RR: 1.63; 95%CI 1.37–1.95), gastrointestinal bleeding (RR: 1.64; 95%CI 1.31–2.06) and intracranial bleeding (RR: 1.61; 95%CI 1.22–2.13) compared to acenocoumarol (Table 3; Figure 3).

**FIGURE 4:**
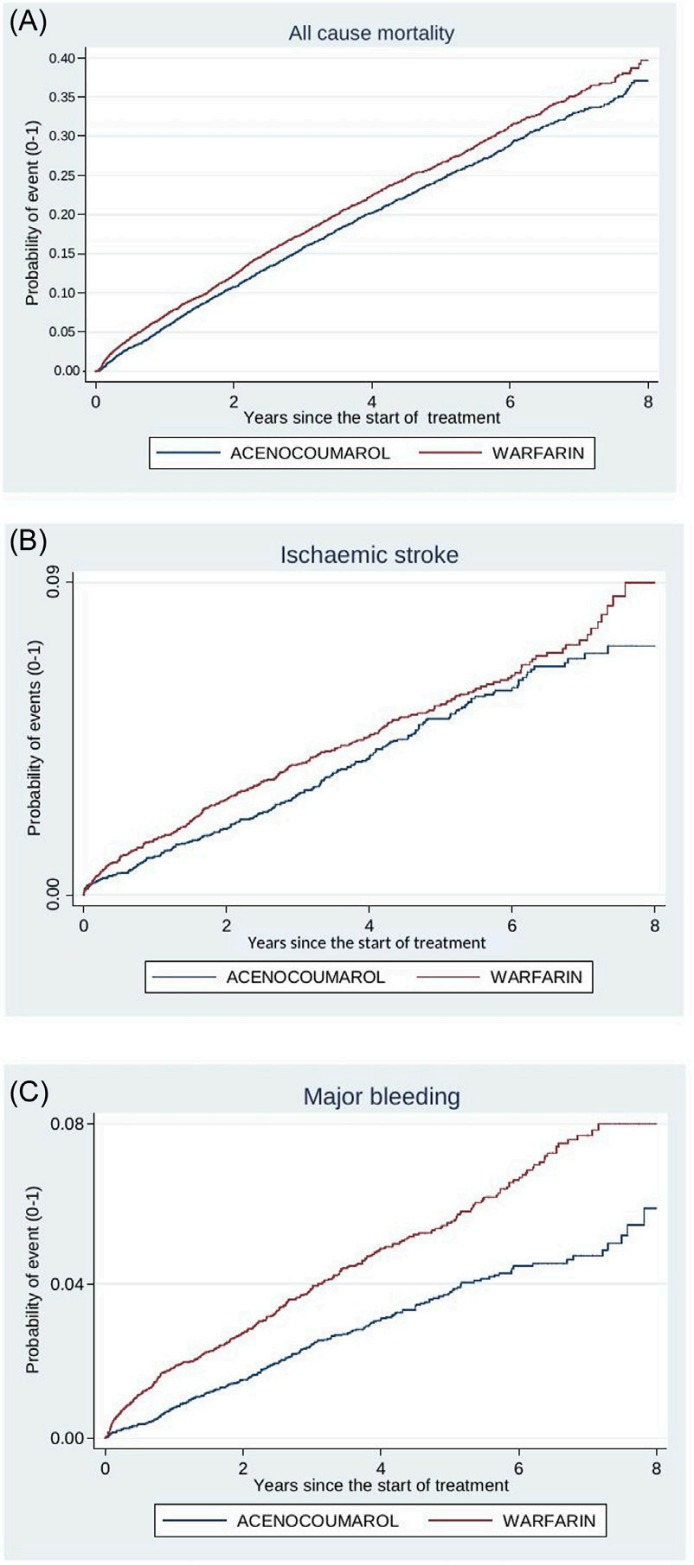
Acenocoumarol vs. warfarin. Crude cumulative incidence or failure curves of **(A)** “all-cause mortality”, **(B)** “ischaemic stroke”, and **(C)** “major bleeding” (intracranial or gastrointestinal bleeding) according to initiated treatment.

**FIGURE 5:**
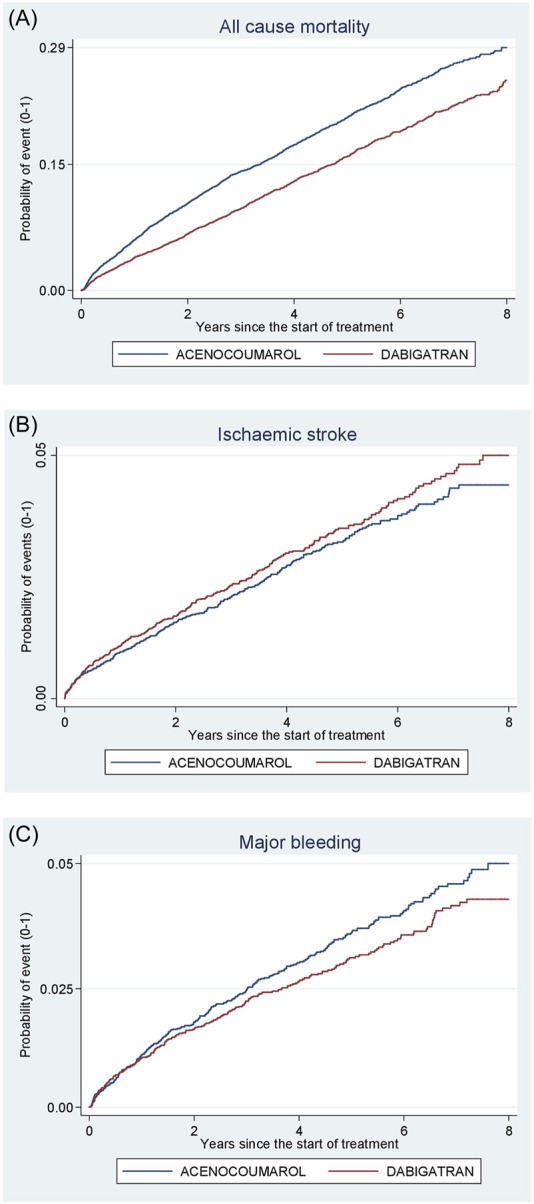
Acenocoumarol vs. dabigatran. Crude cumulative incidence or failure curves of **(A)** “all-cause mortality”, **(B)** “ischaemic stroke”, and **(C)** “major bleeding” (intracranial or gastrointestinal bleeding) according to initiated treatment.

**FIGURE 6:**
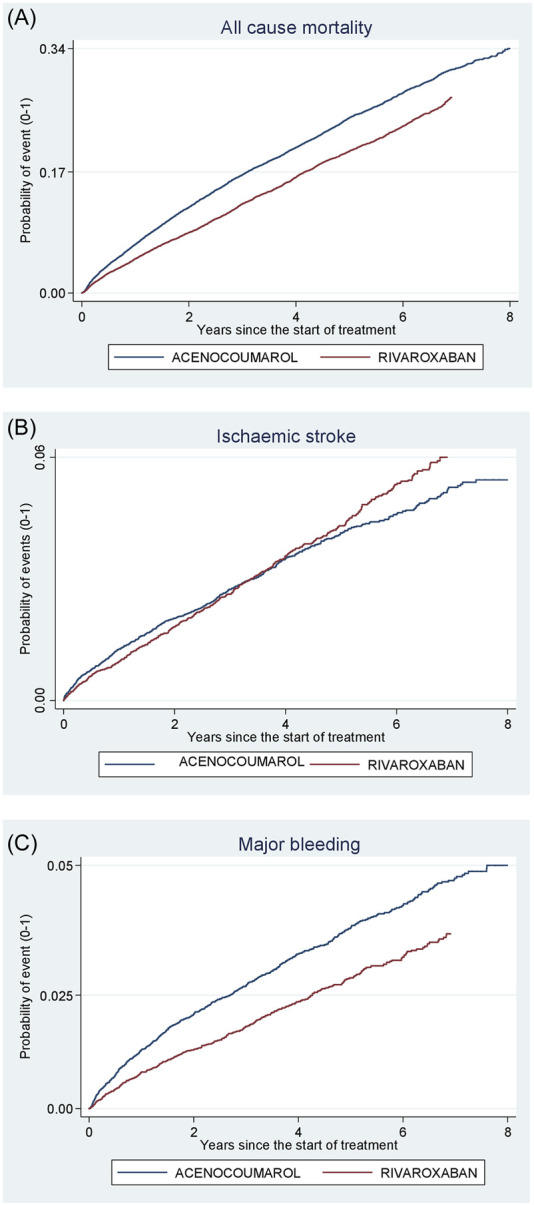
Acenocoumarol vs. rivaroxaban. Crude cumulative incidence or failure curves of **(A)** “all-cause mortality”, **(B)** “ischaemic stroke”, and **(C)** “major bleeding” (intracranial or gastrointestinal bleeding) according to initiated treatment.

**FIGURE 7:**
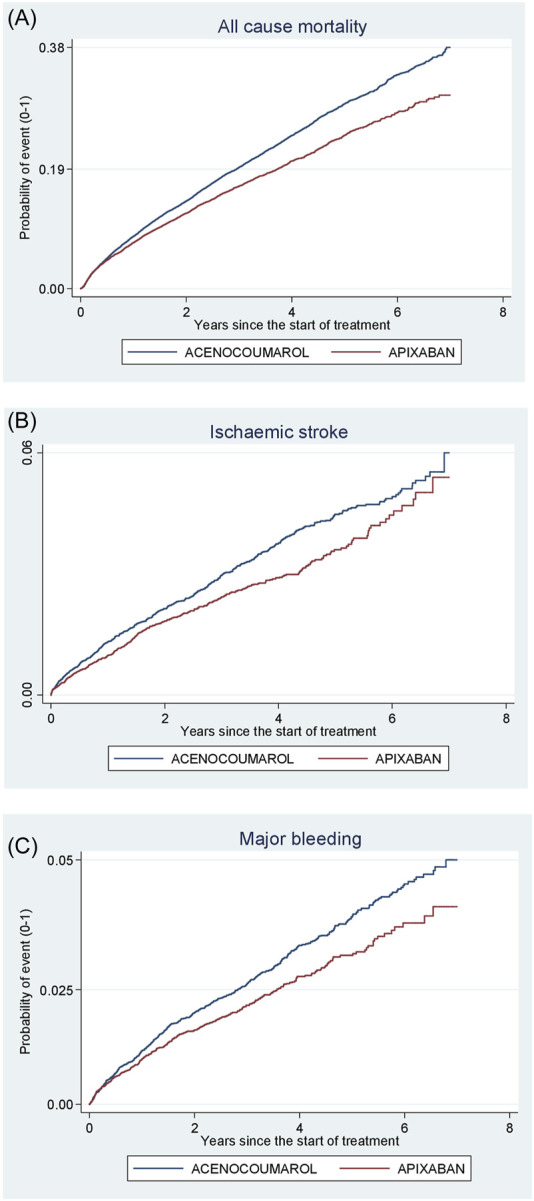
Acenocoumarol vs. apixaban. Crude cumulative incidence or failure curves of **(A)** “all-cause mortality”, **(B)** “ischaemic stroke”, and **(C)** “major bleeding” (intracranial or gastrointestinal bleeding) according to initiated treatment.

**FIGURE 8:**
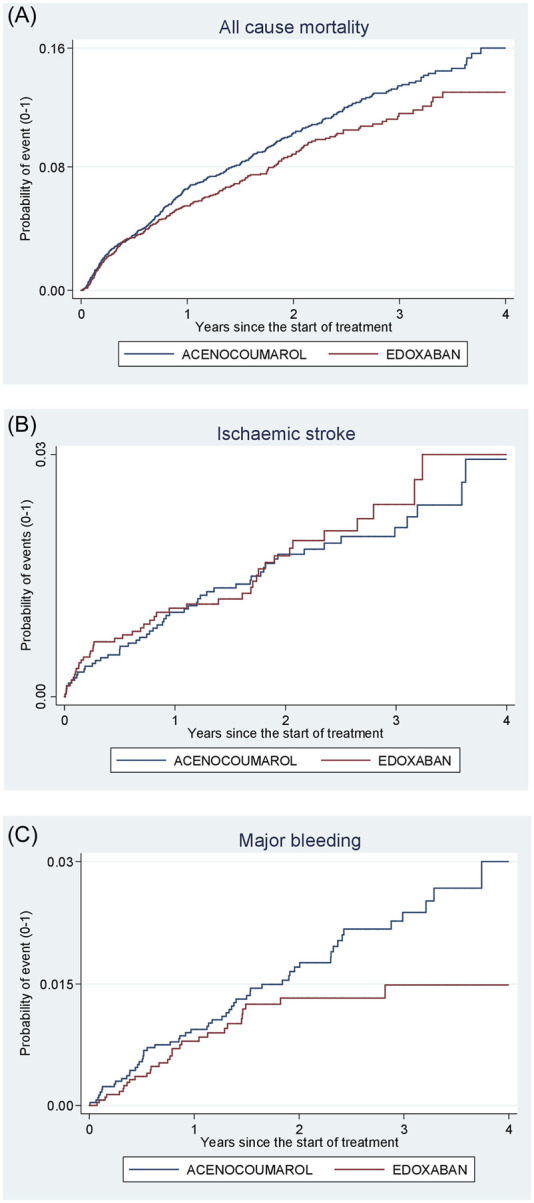
Acenocoumarol vs. edoxaban. Crude cumulative incidence or failure curves of **(A)** “all-cause mortality”, **(B)** “ischaemic stroke”, and **(C)** “major bleeding” (intracranial or gastrointestinal bleeding) according to initiated treatment.

Figures 4–8 show the cumulative incidence (Kaplan-Meier curves) of all-cause mortality, ischaemic stroke and major bleeding (intracranial or gastrointestinal) for each pair of anticoagulants.

A comparison of incidence rates for combined effectiveness and safety by anticoagulant pair is shown in Supplementary Tables S2-S6 **.** Incidence rates for specific event types are shown in Supplementary Tables S7-S11.

### Analysis of other confounding factors

The results obtained using the described methodology (calculation of relative risks, prior PSM, Table 3) are robust, as demonstrated by their concordance with those obtained using multivariate modelling (see Supplementary Table S12) and hazard ratios (HR). This was observed for the combined effectiveness events: RR = 1.11 vs. HR = 1.05 for warfarin; RR = 0.84 vs. HR = 0.82 for dabigatran; RR = 0.85 vs. HR = 0.91 for rivaroxaban; and RR = 0.88 vs. HR = 0.96 for apixaban.

Similar results were obtained for the combined safety events: RR = 1.63 vs. HR = 1.64 for warfarin; RR = 0.88 vs. HR = 0.84 for dabigatran; RR = 0.73 vs. HR = 0.71 for rivaroxaban; and RR = 0.85 vs. HR = 0.87 for apixaban.

These data can be explained by controlling for confounding factors in the models, which had little influence on the outcome. Propensity score matching of groups prior to multivariate analysis reduced the distances between variables. Relative risks and hazard ratios are typically very similar when follow-up times are the same for the entire sample (Martínez-González and Alonso 2008) and in our study, follow-up times were largely homogeneous between pairs after matching cases on propensity score.

### Sensitivity analysis

Consistency and possible differences in outcomes by sex were assessed by performing new analyses of incidence density rates and RR by propensity score-matched groups. The statistically significant reduction in the combined effectiveness risk of ischaemic events was maintained with dabigatran, rivaroxaban and apixaban in males, and with rivaroxaban and apixaban in females. An increased risk of ischaemic stroke was observed with dabigatran in females (Tables 6, 7).

**TABLE 6:** Study in Females: Matched groups of anticoagulants versus acenocoumarol (propensity score matching): Relative risk and 95% confidence intervals for combined effectiveness, combined safety and specific event types.

Type of event during follow-up	Warfarin (n = 3,490)	Dabigatran (n = 3,912)	Rivaroxaban (n = 9,668)	Apixaban^a^ (n = 8,806)	Edoxaban^a^ (n = 2,318)
Effectiveness					
Combined Effectiveness^b^	1.10 (0.97–1.25)	0.89 (0.79–1.01)	0.83 (0.77–0.90)	0.84 (0.77–0.92)	0.99 (0.78–1.26)
Transient ischaemic attack	1.01 (0.67–1.53)	1.06 (0.71–1.59)	1.03 (0.81–1.32)	1.16 (0.86–1.58)	0.84 (0.34–1.97)
Systemic embolism	1.81 (0.46–8.42)	0.72 (0.21–2.26)	1.42 (0.60–3.46)	0.90 (0.29–2.62)	1.34 (0.10–18.55)
Pulmonary embolism	2.06 (0.55–9.37)	0.77 (0.19–2.82)	0.94 (0.45–1.93)	1.29 (0.64–2.57)	0.67 (0.06–4.69)
Ischaemic stroke	1.31 (0.92–1.88)	1.59 (1.11–2.30)	1.21 (0.96–1.52)	0.81 (0.62–1.07)	1.41 (0.73–2.75)
All-cause mortality	1.06 (0.92–1.23)	0.79 (0.68–0.92)	0.75 (0.69–0.82)	0.81 (0.74–0.89)	0.95 (0.71–1.26)
Safety					
Combined Safety^c^	1.95 (1.32–2.94)	0.90 (0.60–1.34)	0.77 (0.60–0.98)	0.92 (0.69–1.22)	0.75 (0.31–1.70)
Gastrointestinal bleeding	1.64 (1.02–2.68)	1.34 (0.80–2.25)	0.86 (0.63–1.15)	0.83 (0.57–1.20)	1.01 (0.37–2.60)
Intracranial bleeding	2.86 (1.35–6.60)	0.43 (0.19–0.89)	0.62 (0.40–0.95)	1.10 (0.69–1.74)	0.22 (0.00–1.84)

^a^ Apixaban and edoxaban are analysed from the year they were marketed: apixaban: 2014–2020; edoxaban: 2017–2020.

^b^ Combined effectiveness: transient ischaemic attack, systemic embolism, pulmonary embolism, ischaemic stroke or all-cause mortality.

^c^ Combined safety: gastrointestinal or intracranial bleeding.

**TABLE 7:** Study in Males: Matched groups of anticoagulants versus acenocoumarol (propensity score matching): Relative risk and 95% confidence intervals for combined effectiveness, combined safety and specific event types.

Type of event during follow-up	Warfarin (n = 4,494)	Dabigatran (n = 6,668)	Rivaroxaban (n = 12,516)	Apixaban ^a^ (n = 9,930)	Edoxaban ^a^ (n = 2,846)
Effectiveness					
Combined Effectiveness^b^	1.17 (1.05–1.30)	0.85 (0.77–0.95)	0.87 (0.81–0.94)	0.91 (0.84–0.99)	0.96 (0.77–1.20)
Transient ischaemic attack	0.87 (0.58–1.30)	0.98 (0.69–1.40)	1.41 (1.08–1.86)	1.36 (1.03–1.81)	0.83 (0.32–2.01)
Systemic embolism	3.68 (0.70–36.33)	2.87 (0.69–16.78)	0.45 (0.13–1.34)	1.12 (0.31–3.90)	—
Pulmonary embolism	0.84 (0.17–3.91)	0.66 (0.24–1.72)	0.73 (0.36–1.45)	1.50 (0.68–3.31)	0.55 (0.05–3.36)
Ischaemic stroke	1.06 (0.78–1.45)	1.18 (0.87–1.60)	0.93 (0.75–1.16)	0.89 (0.68–1.15)	0.81 (0.41–1.57)
All-cause mortality	1.21 (1.08–1.37)	0.79 (0.70–0.89)	0.83 (0.77–0.90)	0.87 (0.80–0.95)	1.00 (0.78–1.28)
Safety					
Combined Safety^c^	1.61 (1.19–2.19)	0.76 (0.56–1.02)	0.70 (0.56–0.88)	0.97 (0.75–1.25)	0.57 (0.26–1.16)
Gastrointestinal bleeding	1.51 (1.00–2.28)	1.37 (0.93–2.05)	0.80 (0.60–1.07)	1.00 (0.72–1.39)	0.78 (0.26–2.15)
Intracranial bleeding	1.73 (1.09–2.81)	0.31 (0.17–0.52)	0.57 (0.38–0.83)	0.92 (0.61–1.39)	0.42 (0.12–1.19)

^a^ Apixaban and edoxaban are analysed from the year they were marketed: apixaban: 2014–2020; edoxaban: 2017–2020.

^b^ Combined effectiveness: transient ischaemic attack, systemic embolism, pulmonary embolism, ischaemic stroke or all-cause mortality.

^c^ Combined safety: gastrointestinal or intracranial bleeding.

The increased risk of TIA associated with rivaroxaban and apixaban remained higher in males. The results of the sensitivity analysis of TIAs with a confirmed hospital diagnosis were consistent with those of the main analysis. These results indicated a higher risk of TIA with apixaban in males (Table 4).

The risk of mortality was lower in both males and females when using dabigatran, rivaroxaban and apixaban (Tables 6, 7).

However, excluding all-cause mortality from the combined effectiveness variable in the sensitivity analysis revealed an increased risk of ischaemic events with dabigatran and rivaroxaban. These results were confirmed in female patients (Table 4).

The subdistribution hazard ratios from the Fine-Gray model of combined effectiveness (excluding mortality) showed the same trend in ischaemic event results when mortality was integrated as a competing risk in survival analysis (Table 5). The results of the combined safety variable also exhibited the same trend after the Fine-Gray model was applied (Table 5).

The statistically significant decreased risk of combined safety was maintained with rivaroxaban in both sexes. The reduced risk of intracranial bleeding was confirmed for dabigatran and rivaroxaban in both sexes (Tables 6, 7). In contrast, warfarin was less effective than acenocoumarol in males and showed a higher risk of major bleeding (intracranial and gastrointestinal bleeding) in both sexes.

## Discussion

Our study was conducted on a large population sample (>150,000 patients) with long-term patient follow-up (real world data).). Using this sample, we compared patient health outcomes for acenocoumarol, (a VKA for which little data from clinical trials or observational studies is available) and warfarin (another VKA). We also estimated the effectiveness and safety of four direct oral anticoagulants (DOACs) compared to acenocoumarol.

Although a clear tendency towards reduced intracranial bleeding was observed with DOACs compared to acenocoumarol, our data did not demonstrate a significant decrease in thromboembolic events when acenocoumarol was used as the reference drug. Warfarin appeared to be less effective and safe than acenocoumarol.

As edoxaban was the most recently marketed DOAC (2017), the mean follow-up time was only 1.6 years and the number of events occurring was small (Supplementary Table S11). There were no statistically significant differences in effectiveness or safety between acenocoumarol and edoxaban. Consequently, firm conclusions cannot be drawn. The SIESTA research team has received funding to extend the follow-up period of the study by 3 years and it is hoped that this will allow us to draw clear conclusions about the effectiveness and safety of edoxaban compared to acenocoumarol.

One factor that lends robustness to our study is that the results for the total population are consistent with the trends observed when the data are stratified by sex. In the latter case, however, some of these results are not statistically significant due to smaller sample sizes when patients are disaggregated.

Different prescribing patterns were observed for oral anticoagulants. Among DOACs, dabigatran was more commonly used among younger patients, whereas apixaban was more frequently prescribed to older patients (over 70 years of age). This pattern of use is consistent with that reported by other investigators (Larsen et al., 2016; Durand et al., 2021; Rodríguez-Bernal et al., 2021). The greater use of dabigatran among younger patients may be related to the recommendation in the package leaflet to reduce the dose (from 150 mg to 110 mg every 12 h) for patients over 80 years of age.

Among patients taking VKAs, those receiving warfarin had a higher incidence of previous heart disease than those treated with acenocoumarol.

Statistical analysis based on PS matching enabled the creation of groups with variables with more similar means or categorical distributions between the different pairs of anticoagulants studied, which allowed us to assess health outcomes in everyday clinical practice using directly comparable groups. This strengthened the conclusions of our study (Ciminata et al., 2022).

### DOACs vs. acenocoumarol: effectiveness

No clear reduction in thromboembolic risk was observed with the use of any DOAC compared to acenocoumarol. The results of the combined effectiveness analysis were significantly influenced by the high proportion of deaths among the total number of first effectiveness events recorded. This led to different outcomes depending on whether all-cause mortality was included in the combined outcome. When all-cause mortality was excluded from the combined effectiveness assessment, dabigatran and rivaroxaban were associated with an increased risk of ischaemic events (at least 2% and 5%, respectively, when considering the lower limit of the confidence interval).

We found no immediate explanation for the increased risk of TIA (at least 1%) alongside the reduced risk of ischaemic stroke (at least 2%) with apixaban. Rivaroxaban was also associated with an increased risk of TIA (at least 4%). This slight increase in the risk of TIA was unexpected, given that DOACs were thought to be as effective, if not more so, than VKAs (Rodríguez-Bernal et al., 2021). One possible explanation for these results may be that the TIA diagnoses were extracted not only from hospital databases but also from notes made by primary care physicians. In the absence of diagnostic confirmation, the incidence of TIA may have been overestimated. Many patients presenting with disorientation and focal neurological signs may have been diagnosed as possible TIA in primary care and referred to hospital for diagnostic confirmation. However, focal neurological signs, disorientation, or consciousness deficit may disappear without being confirmed by neurological imaging tests. This would suggest that the transient neurological condition was not thrombotic in origin. The results of our sensitivity analysis, which included only TIAs with confirmed hospital diagnosis, were consistent with those of the main analysis, and showed a small increase in the risk of TIA for rivaroxaban and apixaban (at least 1%).

These data are inconsistent with those of Rodríguez-Bernal et al. (2021), who found no significant overall difference in the incidence of TIA between the three DOACs evaluated (dabigatran, rivaroxaban, and apixaban) compared to acenocoumarol.

In our study, apixaban was the only DOAC that showed a reduced risk of ischaemic stroke compared to acenocoumarol. The other DOACs showed a trend towards an increased risk of ischaemic stroke, though this was not statistically significant. These results are consistent with those of Rodríguez-Bernal et al. (2021).

In a Spanish cohort of 2,178 patients, Anguita et al. (2020) observed a trend towards a lower incidence of stroke, systemic embolism, and all-cause mortality with DOACs compared to acenocoumarol, although the differences were not statistically significant. The study’s substantial data set allowed for statistically significant but small RR values close to 1.

In our study, all-cause death was the most common event recorded, with 29,475 cases corresponding to 19.5% of those under follow-up and 61.8% of total events recorded, which is consistent with the results obtained by Rodríguez-Bernal et al. (2021), in which mortality was the most frequently recorded event (accounting for around 70% of events). Three DOACs (dabigatran, rivaroxaban, and apixaban) showed a decreased risk of all-cause mortality compared to acenocoumarol. These data contradict those obtained by Rodríguez-Bernal et al. (2021) and Anguita et al. (2020) in similar populations, who found no difference in mortality for DOACs compared to acenocoumarol. The differences in our study may be due to the longer follow-up period (8 years) and the higher number of all-cause deaths. Furthermore, the statistical analysis used by Rodríguez-Bernal et al. (2021) was based on (inverse probability weighting (IPW), whereas our study used propensity score matching. Nevertheless, when other investigators (Austin, 2011; Ciminata et al., 2022) used these two analytical models to evaluate DOACs in patients with AF, they found that there were no statistically significant differences.

With regard to mortality (which accounted for almost one-fifth of the patients under follow-up), we emphasise the systematic and thorough collection of death data from the official statistical sources of the Autonomous Community of Andalusia. We do not know which factors had a greater impact on the risk of mortality in patients with AF. Although this study considered many demographic and health-related variables, it would have been impossible to record every factor or describe the complex interplay of causes implicated in such a wide-ranging issue as the probability of death. The use of medications was just one of the variables involved.

The paired analysis by sex revealed a mortality trend similar to that observed in the overall sample. Rodríguez-Bernal et al. (2021) also observed a reduced risk of mortality among males taking dabigatran compared to acenocoumarol, but not among females.

### DOACs vs. acenocoumarol: safety

The comparative safety evaluation showed that the overall risk of bleeding events was significantly lower with rivaroxaban and apixaban than with acenocoumarol. All four DOACs evaluated against acenocoumarol showed a significant decrease in the incidence of intracranial bleeding. However, rivaroxaban was the only DOAC to significantly reduce the incidence of gastrointestinal bleeding, while dabigatran was the least safe DOAC, increasing the risk.

These results differ somewhat from those of Rodríguez-Bernal et al. (2021), who found no differences in gastrointestinal bleeding. However, they observed a reduction in intracranial bleeding with dabigatran and rivaroxaban compared to acenocoumarol. Anguita et al. (2020). Observed a trend towards a lower incidence of severe bleeding with DOACs compared to VKAs (primarily acenocoumarol), although it was not statistically significant.

Our results are partly consistent with those obtained by van den Ham et al. (2021), who conducted a pooled analysis of databases from four European countries and six Canadian provinces. They found that apixaban showed a lower risk of gastrointestinal bleeding, whereas dabigatran and rivaroxaban showed an increased risk compared to VKAs. They also confirmed a decreased risk of intracranial bleeding for all DOACs compared to VKAs.

With regard to the sensitivity analysis by sex, our results are consistent with those obtained in the primary analysis. The risk of intracranial bleeding is consistent with that obtained by Rodríguez-Bernal et al. (2021), who found a statistically significant reduction for dabigatran and rivaroxaban in females, and for dabigatran in males, compared to acenocoumarol.

As some investigators have pointed out (Ingason et al., 2021), the effectiveness and safety of DOACs in real-world practice is greatly influenced by differences in patient adherence. Although DOACs have similar half-lives, dabigatran and apixaban require twice-daily dosing. Durand et al. (2021) have suggested that this could explain their observation of an increase in ischaemic and bleeding events with rivaroxaban at the recommended once-daily dose, since this is associated with higher peak plasma concentrations that decline progressively after dosing. However, our results do not support this theory. While it could be argued that lower adherence to apixaban (which requires two daily doses) might explain the trend towards an increase in the risk of TIA observed in our study, we could find no justification for the same increase in TIA with rivaroxaban (with a once-daily dose) nor for the reduced risk of ischaemic stroke with apixaban. As we have not conducted a comparative study between different DOACs or evaluated patient adherence, we are unable to draw conclusions about the effect of the dosing regimen on health outcomes.

The RE-LY study (Connolly et al., 2009) revealed that the proportion of patients who discontinued dabigatran treatment due to gastrointestinal complications was three times higher than with warfarin. These results could also explain those obtained in our study, in which dabigatran was the only DOAC to show an increased risk of major gastrointestinal bleeding.

It is important to note that we did not compare the effectiveness and safety of DOACs with patients receiving treatment with VKA with good anticoagulation control (high time in therapeutic range, TTR), which may have overestimated the effect of DOACs in the study. Wallentin et al. (2010) investigated the primary and secondary outcomes of the RE-LY trial (Connolly et al., 2009) in relation to the mean TTR in each centre in warfarin patients. They concluded that dabigatran offered greater advantages for all vascular events, non-haemorrhagic events, and mortality at sites with poor INR control than at sites with good INR control in warfarin patients. Joosten et al. (2024) conducted a clinical trial in frail patients with AF (aged ≥75 years with a Groningen Frailty Indicator score ≥3) and concluded that switching from INR–guided VKA treatment to a DOAC in frail older patients with AF was associated with more bleeding complications compared with continuing VKA treatment, without reducing thromboembolic complications. Doni et al. (2024) conducted a systematic review of randomized controlled trials on DOACs to assess the safety of long-term intake of DOACs in older adults with AF. They found that elderly patients (aged 85 years and older) who use DOACs may be at an increased risk of major or clinically relevant bleeding events compared to those who use VKAs.

Based on these considerations, our study’s findings would support the recommendation in the 2024 European Society of Cardiology Guidelines (Van Gelder et al., 2024) that, for older patients aged ≥75 years with polypharmacy, who are clinically stable, and on therapeutic VKA, maintaining VKA treatment rather than switching to a DOAC may be considered in order to minimise bleeding risk.

### Warfarin vs. acenocoumarol

Warfarin has been the traditional comparator drug used in clinical trials and observational studies against the DOACs (Connolly et al., 2009; Granger et al., 2011; Patel et al., 2011; Giugliano et al., 2013; Larsen et al., 2016; Nielsen et al., 2017; Domek et al., 2020). The results of our study revealed statistically significant and clinically relevant differences in both effectiveness and safety between the two VKA drugs. Compared to acenocoumarol, warfarin was associated with an increased risk of systemic embolism (by at least 16%), an increased risk of all-cause mortality (by 5%), and an increased risk of major bleeding events (by 37%), taking the lower limit of the confidence interval for the relative risk (Table 3).

These data suggest that conclusions drawn from studies using warfarin as the reference drug should not be automatically extrapolated to acenocoumarol.

### Strengths

Firstly, the large sample size and long follow-up period of our study allow extrapolation to the general population, providing high external validity.

Secondly, our study provides a comparative evaluation of two VKAs with different half-lives, acenocoumarol and warfarin, in a routine clinical practice setting. This may explain the differing outcomes obtained when comparing them with DOACs and with each other. We also emphasise that the study was conducted by a multidisciplinary team and that there were no conflicts of interest with the pharmaceutical industry.

A third strength of our study is the systematic and thorough collection of mortality data from official records, which contributes to the dependability and reliability of the data. This approach sets our study apart from others, as demonstrated by the numbers and proportions of event types included.

Fourth, our methodology enabled us to select various sources, types of data, as well as essential variables for use in future studies, and to assess the usefulness of each variable in terms of reliability, consistency, and proportion of missing data. In addition, we standardised and normalised disease diagnoses found in various classifications (ICD-9, ICD-10, and the International Classification of Primary Care) and developed integrated scales and clinical evaluations of patients based on different demographic information, comorbidities, use of resources, etc.

Fifthly, a dual approach was adopted using R and Stata applications. This was done firstly to ensure more robust results, and secondly to use the most efficient, user-friendly, and widely-recognised instructions and utilities. We consider the establishment of a methodology and essential information for future drug evaluation studies, including those from other therapeutic groups within our organisation, to be a significant achievement. Consequently, we have preserved and clearly explained all syntax and commands for each step. With our large and diverse multidisciplinary team, we envisage this study laying the foundation of a series of prioritised studies to be developed over time. Any necessary improvements and specific issues related to each study can be incorporated as needed.

Finally, a methodology was chosen that enabled us to transition from a purely observational cohort study of patients to a quasi-experimental study emulating a clinical trial in the comparative evaluation of six oral anticoagulants.

### Limitations

The first limitation is the risk of bias inherent in any observational study. We attempted to control for this by performing a propensity score-matched cohort analysis (PSM).

A second limitation of observational studies is the possibility of missing data in the patients’ clinical records. In our study, we extracted each patient’s clinical information from several databases (primary care, hospital, outpatient, emergency), in order to facilitate the collection of essential variables for the analysis.

Thirdly, we did not include all relevant information, as not all patients had it available. This included information such as smoking, alcohol consumption, diet or the patient’s index of dependence on others for performing basic activities of daily living (Barthel test). Therefore, as with any study, we cannot rule out the possibility that these and other unaccounted for variables may have influenced the results.

Fourthly, there is a possible risk of selection bias for oral anticoagulants, since DOACs require prior authorisation and their dosage depends on the patient’s renal function. Depending on the referring hospital, there may also be differences in the choice of VKA. This issue was addressed through the implementation of propensity score-matched cohort analysis (PSM).

Fifthly, we operated on the assumption that the medication dispensed was the same as the one taken by the patient, as determined by other investigators (Rosa et al., 2018; Durand et al., 2021; Ingason et al., 2021; Lip et al., 2021). However, discrepancies between the prescribed medication and the medication actually taken cannot be entirely ruled out in some cases.

Sixthly, for patients who changed medication during the follow-up period (“per-protocol analysis”), we did not conduct a health outcomes analysis based on the most recent OAC the patient was taking when the event was recorded. However, our analysis using the intention-to-treat approach is consistent with standard methods employed in other observational studies (Larsen et al., 2016; Nielsen et al., 2017; Durand et al., 2020; Rodríguez-Bernal et al., 2021; Lau et al., 2022) and in clinical trials (Connolly et al., 2009; Granger et al., 2011; Patel et al., 2011; Giugliano et al., 2013). In the near future, we will perform a complementary pre-treatment analysis, examining outcomes based on the treatment that patients were receiving immediately before an effectiveness or safety event occurred, rather than looking only at their initial treatment assignment.

Seventhly, we did not consider an analysis of low-dose versus standard dose of DOACs.

We also did not assess the relative efficiency or cost implications of the switch in prescription practice from VKA to DOACs. These are open questions for future analysis.

Eighthly, it was not possible to access the INR registry databases in order to evaluate the proper control of patients undergoing VKA treatment. We are aware that poor INR monitoring in acenocoumarol users may result in an artificial inflation of the risks of bleeding or mortality, thereby favouring DOACs. Therefore, our findings should be interpreted with caution in light of this limitation. This aspect will be the subject of future investigations by our research team.

Ninthly, we did not take into account the time that elapsed between the AF diagnosis and the initiation of anticoagulant therapy. This may contribute to potential confounding by disease duration, which could differ systematically across drug groups. Nevertheless, the study exclusively included new users of oral anticoagulants, defined as patients who had not used OACs during the 12 months preceding their enrolment in the study, regardless of any prior use.

## Conclusion

Our study has shown that, in general, there are some differences in the effectiveness and safety of DOACs compared to acenocoumarol. Due to a lack of sufficient data, a clear evaluation of edoxaban could not be made. The other three DOACs (dabigatran, rivaroxaban and apixaban) did not demonstrate a significant reduction in ischaemic events.

The three DOACs appear to be safer, with a lower incidence of severe bleeding compared to acenocoumarol, except for dabigatran, which achieved good results in the incidence of intracranial bleeding but had a higher incidence of gastrointestinal bleeding compared to acenocoumarol. These results are supported by the analysis of paired data disaggregated by sex. The values observed for both sexes are comparable to and consistent with the global data. Warfarin was less effective and less safe in comparison with acenocoumarol.

The results provide evidence from real-world clinical practice which needs to be confirmed in well-designed clinical trials involving acenocoumarol. Both types of study, RWD and clinical trials, complement each other and allow us to expand our knowledge of treatments and determine the role of new drugs in the therapeutic arsenal. The focus of our future research will be on analysing patient subgroups with certain comorbidities (e.g., diabetes, renal insufficiency), older patients, with inadequate anticoagulation (time in therapeutic range) for VKA drugs, and the use of low-dose DOACs compared to acenocoumarol. Our results are expected to be of great value when updating clinical practice guidelines in healthcare systems. This information will be important for clinicians in selecting and personalising treatments for patients with atrial fibrillation.

## Data Availability

Data cannot be shared publicly because they are based on the Andalusian Health Population Database. Data are available from the Subdirección Técnica Asesora de Gestión de la Información, Andalusian Health System, Seville, Spain, for researchers who meet the criteria for access to confidential data.
