# Supplementary material for: Comparative clinical outcomes of acenocoumarol versus direct oral anticoagulants (DOACs) and warfarin in patients with atrial fibrillation: real-world-evidence (SIESTA-A study)
Source: Front Pharmacol. 2025 Aug 1;16:1548298. doi: 10.3389/fphar.2025.1548298 (PMC12354484; doi:10.3389/fphar.2025.1548298)
Supplement: Supplementary file 1 [file Table1.docx]

**SUPPLEMENTARY TABLES**

| **Supplementary Table 1A.** **Differences between selected quantitative variables are expressed in real units, as specified in the characteristics of each variable: A) Matched groups, B) Differences between complete cohorts.** | | | | | |
| --- | --- | --- | --- | --- | --- |
|  |  |  |  |  |  |
|  | 1. **A) DIFFERENCES BETWEEN PROPENSITY SCORE-MATCHED GROUPS** | | |  |  |
| **Characteristics** | **ACENOCOUMAROL WARFARIN** | **ACENOCOUMAROL**  **DABIGATRAN** | **ACENOCOUMAROL**  **RIVAROXABAN** | **ACENOCOUMAROL**  **APIXABAN** | **ACENOCOUMAROL**  **EDOXABAN** |
|  | (n=7,471 pairs) | (n=9,712 pairs) | (n=20,874 pairs) | (n=23,506 pairs) | (n=7,877 pairs) |
| **Age (years), mean difference** | -1.00 | 0.00 | 1.00 | 0.00 | 0.00 |
| **CHADS-VASC score (between 0-9), mean difference** | -0.14 | 0.07 | 0.13 | 0.04 | 0,02 |
| **HAS-BLED score (between 0-7),**  **mean difference** | -0.05 | 0.04 | 0.06 | 0.02 | 0,01 |
| **Hospital emergency (per 100 person-years of follow-up), mean difference** | -11.30 | 7.40 | -0.30 | 5.60 | -6.00 |
| **Hospital admission (per 100 person-years of follow-up), mean difference** | -1.20 | -0.20 | 3.00 | 3.20 | 3.30 |
| 1. **DIFFERENCES BETWEEN COMPLETE COHORTS (Total, n= 150,949)** | | | | | |
| **Characteristics** | **ACENOCOUMAROL WARFARIN** | **ACENOCOUMAROL**  **DABIGATRAN** | **ACENOCOUMAROL**  **RIVAROXABAN** | **ACENOCOUMAROL**  **APIXABAN** | **ACENOCOUMAROL**  **EDOXABAN** |
| **Age (years), mean difference** | 1.00 | 3.00 | 2.00 | 0.00 | 2.00 |
| **CHADS-VASC score (between 0-9),**  **mean difference** | 0.00 | 0.40 | 0.30 | 0.00 | 0.30 |
| **HAS-BLED score (between 0-7),**  **mean difference** | 0.10 | 0.30 | 0.20 | 0.10 | 0.20 |
| **Hospital emergency (per 100 person-years of follow-up), mean difference** | 10.60 | 19.70 | 17.50 | 17.20 | 27.30 |
| **Hospital admission (per 100 person-years of follow-up), mean difference** | -4.80 | 4.50 | 5.50 | 3.60 | 10.90 |
| **Supplementary Table 1B. Standardized mean differences (SMDs) between selected quantitative variables: A) Matched groups, B) Differences between complete cohorts.** | | | | | |
|  |  |  |  |  |  |
|  | 1. **DIFFERENCES BETWEEN PROPENSITY SCORE-MATCHED GROUPS** | | |  |  |
| **Characteristics** | **ACENOCOUMAROL WARFARIN** | **ACENOCOUMAROL**  **DABIGATRAN** | **ACENOCOUMAROL**  **RIVAROXABAN** | **ACENOCOUMAROL**  **APIXABAN** | **ACENOCOUMAROL**  **EDOXABAN** |
|  | (n=7,471 pairs) | (n=9,712 pairs) | (n=20,874 pairs) | (n=23,506 pairs) | (n=7,877 pairs) |
| **Age, mean** | -0.12 | 0.07 | 0.09 | 0.00 | -0.03 |
| **CHADS-VASC score, mean** | -0.08 | 0.04 | 0.08 | 0.02 | 0.01 |
| **HAS-BLED score, mean** | -0.05 | 0.04 | 0.06 | 0.02 | 0.00 |
| **Hospital emergency, mean** | 0.09 | -0.04 | 0.03 | 0.04 | -0.05 |
| **Hospital admission, mean** | -0.03 | -0.01 | 0.07 | 0.08 | 0.09 |
| 1. **DIFFERENCES BETWEEN COMPLETE COHORTS (Total, n= 150,949)** | | | | | |
| **Characteristics** | **ACENOCOUMAROL WARFARIN** | **ACENOCOUMAROL**  **DABIGATRAN** | **ACENOCOUMAROL**  **RIVAROXABAN** | **ACENOCOUMAROL**  **APIXABAN** | **ACENOCOUMAROL**  **EDOXABAN** |
| **Age, mean** | 0.09 | 0.29 | 0.19 | -0.02 | 0.17 |
| **CHADS-VASC score, mean** | 0.01 | 0.25 | 0.18 | 0.00 | 0.20 |
| **HAS-BLED score, mean** | 0.05 | 0.25 | 0.21 | 0.03 | 0.19 |
| **Hospital emergency, mean** | 0.09 | 0.17 | 0.15 | 0.15 | 0.23 |
| **Hospital admission, mean** | -0.12 | 0.10 | 0.13 | 0.08 | 0.26 |

**Supplementary Table 2. Acenocoumarol vs warfarin: Comparison of incidence density rates by type of combined event and selected variables (paired data). Events occurring per 1,000 person-years of follow-up are shown.**

|  | EFFECTIVENESS | | SAFETY | |
| --- | --- | --- | --- | --- |
| Sex  Women  Men  Age (years)  40-59  60-69  70-79  80-89  90-112  Pharmaceutical co-payment status  Employed  Exempt from payment  Pensioners  Hospital admissions  Low level  Medium level  High level  CHADS-VASC  Low risk  Medium risk  High risk  HAS BLED  Low risk  Medium risk  High risk | Acenocoumarol  82.69  77.36  24.97  41.12  74.44  162.22  315.16  26.13  24.97  112.55  28.21  46.5  172.91  22.82  50.12  128.21  36.17  81.7  186.51 | Warfarin  85.38  92.18  25.19  39  81.1  157  338.1  19.95  27.05  127.03  31.54  50.74  200.05  33.93  56.95  130.66  44.82  89.77  182.14 | Acenocoumarol  6.79  8.19  4.03  7.01  8.28  12.79  19.72  4.19  5.49  8.96  0.11  5.53  17.23  3.17  5.78  10.69  4.2  7.81  14.43 | Warfarin  11.34  13.34  2.95  7.04  8.48  20.71  13.18  3.79  8.04  15.36  0.12  7.21  31.43  5.01  10.47  15.8  5.54  12.33  28.32 |

**Supplementary Table 3. Acenocoumarol vs dabigatran: Comparison of incidence density rates by type of combined event and selected variables (paired data). Events occurring per 1,000 person-years of follow-up are shown.**

|  | EFFECTIVENESS | | SAFETY | |
| --- | --- | --- | --- | --- |
| Sex  Women  Men  Age  40-59  60-69  70-79  80-89  90-112  Pharmaceutical co-payment status  Employed  Exempt from payment  Pensioners  Hospital admissions  Low level  Medium level  High level  CHADS-VASC  Low risk  Medium risk  High risk  HAS BLED  Low risk  Medium risk  High risk | Acenocoumarol  65.07  62.57  18.93  29.66  60.09  127.63  226.10  16.27  21.77  92.93  31.63  46.88  177.41  19.39  47.33  103.66  29.69  70.60  137.18 | Dabigatran  53.95  53.03  17.98  27.74  50.30  112.50  195.17  17.07  18.99  77.67  21.91  39.94  148.4  17.95  40.13  88.65  24.51  59.97  117.14 | Acenocoumarol  6.87  7.96  3.01  5.8  7.95  11.44  10.57  3.12  5.16  9.43  0.42  7.68  24.52  3.24  6.92  10.14  4.25  7.97  16.86 | Dabigatran  6.81  6.43  0.86  4.39  7.88  10.59  21.68  1.48  4.89  8.39  0.36  4.87  22.63  1.25  5.64  10.49  2.78  7.31  16.78 |

**Supplementary Table 4. Acenocoumarol vs Rivaroxaban: Comparison of incidence density rates by type of combined event and selected variables (paired data). Events occurring per 1,000 person-years of follow-up are shown.**

|  | EFECTIVIDAD | | seguridad | |
| --- | --- | --- | --- | --- |
| Sex  Women  Men  Age  40-59  60-69  70-79  80-89  90-112  Pharmaceutical co-payment status  Employed  Exempt from payment  Pensioners  Hospital admissions  Low level  Medium level  High level  CHADS-VASC  Low risk  Medium risk  High risk  HAS BLED  Low risk  Medium risk  High risk | **Acenocoumarol**  77.07  74.60  22.70  32.49  66.69  137.54  236.92  19.06  24.76  107.04  35.29  53.77  193.23  20.52  52.54  115.91  32.14  81.99  157.97 | **Rivaroxaban**  63.42  65.43  18.78  27.39  55.47  126.08  271.30  17.02  21.16  93.29  27.29  48.31  167.21  20.59  44.09  99.56  29.10  68.93  137.21 | **Acenocoumarol**  7.57  8.84  3.82  5.23  8.85  11.38  14.09  3.06  5.90  10.01  0.57  8.14  23.5  3.86  7.23  10.58  4.59  8.22  20.2 | **Rivaroxaban**  5.70  6.25  2.19  3.81  5.83  10.19  8.25  2.10  5.46  6.82  0.00  5.88  17.52  2.01  4.43  8.80  2.41  6.26  14.87 |

**Supplementary Table 5. Acenocoumarol vs Apixaban: Comparison of incidence density rates by type of combined event and selected variables (paired data). Events occurring per 1,000 person-years of follow-up are shown.**

|  | EFFECTIVENESS | | SAFETY | |
| --- | --- | --- | --- | --- |
| Sex  Women  Men  Age  40-59  60-69  70-79  80-89  90-112  Pharmaceutical co-payment status  Employed  Exempt from payment  Pensioners  Hospital admissions  Low level  Medium level  High level  CHADS-VASC  Low risk  Medium risk  High risk  HAS BLED  Low risk  Medium risk  High risk | Acenocoumarol  95.28  93.11  25.64  40.18  79.17  155.39  297.41  22.80  28.57  139.03  40.27  67.25  221.22  25.36  65.05  135.55  37.91  97.62  185.34 | Apixaban  79.27  85.55  20.39  32.32  59.14  146.16  289.76  19.98  23.24  124.23  27.22  62.68  204.92  21.42  55.10  120.60  34.87  84.78  167.01 | Acenocoumarol  7.73  8.83  2.92  5.87  8.95  10.56  12.57  2.57  5.80  10.14  0.52  7.38  22.71  3.33  7.40  10.24  4.13  8.22  17.95 | Apixaban  6.73  8.29  3.09  3.54  7.34  11.74  11.54  3.89  5.68  9.01  0.00  6.09  21.18  2.34  6.36  9.76  3.25  7.34  17.97 |

**Supplementary Table 6. Acenocoumarol vs Edoxaban: Comparison of incidence density rates by type of combined event and selected variables (paired data). Events occurring per 1,000 person-years of follow-up are shown.**

|  | EFFECTIVENESS | | SAFETY | |
| --- | --- | --- | --- | --- |
| Sex  Women  Men  Age  40-59  60-69  70-79  80-89  90-112  Pharmaceutical co-payment status  Employed  Exempt from payment  Pensioners  Hospital admissions  Low level  Medium level  High level  CHADS-VASC  Low risk  Medium risk  High risk  HAS BLED  Low risk  Medium risk  High risk | **Acenocoumarol**  67.47  68.10  21.62  41.83  53.05  139.38  213.13  25.38  25.23  108.24  21.55  69.36  241.20  23.12  48.29  113.30  23.58  71.82  222.18 | **Edoxaban**  68.95  60.12  16.85  22.12  53.41  136.04  230.98  10.59  24.46  100.71  16.81  72.62  196.18  13.44  45.09  110.66  26.93  70.21  153.84 | **Acenocoumarol**  6.14  7.02  2.17  5.14  7.84  9.74  6.29  2.46  5.46  8.09  0.50  7.91  24.82  2.69  6.51  8.67  2.97  7.62  12.15 | **Edoxaban**  7.16  6.30  2.87  3.38  7.55  11.35  5.69  2.29  5.41  8.44  0.00  6.61  27.62  3.64  4.47  10.66  3.04  6.75  21.96 |
|  |  |  |  |  |

| **Supplementary Table 7: Acenocoumarol vs warfarin. Comparison of incidence density (/1,000 person-years of follow-up) by anticoagulant pairs according to event type. Number of pairs compared = 7,471 (8 years of follow-up between 2013-2020).** | | | | | |
| --- | --- | --- | --- | --- | --- |
|  | |  |  |  |  |
| **ACENOCOUMAROL - Warfarin** | **ANTICOAGULANT** | **Events**  **(n)** | **Incidence Rates^1^** | **Relative**  **Risk** | **95%CI** |
| **Effectiveness** |  |  |  |  |  |
| **Combined Effectiveness** | ACENOCOUMAROL | 2,139 | 79.72 | 1 |  |
|  | Warfarin | 2,323 | 88.86 | 1.11 | 1.05-1.18 |
|  |  |  |  |  |  |
| All-cause mortality | ACENOCOUMAROL | 1,637 | 61.01 | 1 |  |
|  | Warfarin | 1,790 | 68.47 | 1.12 | 1.05-1.20 |
|  |  |  |  |  |  |
| Transient ischaemic attack | ACENOCOUMAROL | 205 | 7.64 | 1 |  |
|  | Warfarin | 179 | 6.85 | 0.9 | 0.73-1.09 |
|  |  |  |  |  |  |
| Systemic embolism | ACENOCOUMAROL | 17 | 0.63 | 1 |  |
|  | Warfarin | 34 | 1.30 | 2.05 | 1.16-3.76 |
|  |  |  |  |  |  |
| Pulmonary embolism | ACENOCOUMAROL | 21 | 0.78 | 1 |  |
|  | Warfarin | 22 | 0.84 | 1.08 | 0.59-1.97 |
|  |  |  |  |  |  |
| Ischaemic stroke | ACENOCOUMAROL | 260 | 9.69 | 1 |  |
|  | Warfarin | 298 | 11.4 | 1.18 | 1-1.39 |
|  |  |  |  |  |  |
| **Safety** |  |  |  |  |  |
| **Combined Safety** | ACENOCOUMAROL | 215 | 7.60 | 1 |  |
|  | Warfarin | 341 | 12.40 | 1.63 | 1.37-1.95 |
|  |  |  |  |  |  |
| Gastrointestinal bleeding | ACENOCOUMAROL | 128 | 4.50 | 1 |  |
|  | Warfarin | 204 | 7.30 | 1.64 | 1.31-2.06 |
|  |  |  |  |  |  |
| Intracranial bleeding | ACENOCOUMAROL | 87 | 3.00 | 1 |  |
|  | Warfarin | 137 | 4.80 | 1.61 | 1.22-2.13 |

^1^ Incidence rates: number of events per 1,000 person-years of follow-up.

| **Supplementary Table 8: Acenocoumarol vs Dabigatran. Comparison of incidence density (/1,000 person-years of follow-up) by anticoagulant pairs according to event type. Number of pairs compared = 9,712 (8 years of follow-up between 2013-2020).** | | | | | |
| --- | --- | --- | --- | --- | --- |
|  | | | |  |  |
| **ACENOCOUMAROL - Dabigatran** | **ANTICOAGULANT** | **Events**  **(n)** | **Incidence Rates^1^** | **Relative**  **Risk** | **95%CI** |
| **Effectiveness** |  |  |  |  |  |
| **Combined Effectiveness** | ACENOCOUMAROL | 2,463 | 63.61 | 1 |  |
|  | Dabigatran | 1,934 | 53.43 | 0.84 | 0.79-0.89 |
|  |  |  |  |  |  |
| All-cause mortality | ACENOCOUMAROL | 1,928 | 49.79 | 1 |  |
|  | Dabigatran | 1,385 | 38.26 | 0.77 | 0.72-0.82 |
|  |  |  |  |  |  |
| Transient ischaemic attack | ACENOCOUMAROL | 206 | 5.32 | 1 |  |
|  | Dabigatran | 222 | 6.13 | 1.15 | 0.95-1.39 |
|  |  |  |  |  |  |
| Systemic embolism | ACENOCOUMAROL | 18 | 0.46 | 1 |  |
|  | Dabigatran | 21 | 0.58 | 1.25 | 0.66-2.37 |
|  |  |  |  |  |  |
| Pulmonary embolism | ACENOCOUMAROL | 33 | 0.85 | 1 |  |
|  | Dabigatran | 19 | 0.52 | 0.62 | 0.34-1.07 |
|  |  |  |  |  |  |
| Ischaemic stroke | ACENOCOUMAROL | 278 | 7.18 | 1 |  |
|  | Dabigatran | 288 | 7.96 | 1.11 | 0.94-1.31 |
|  |  |  |  |  |  |
| **Safety** |  |  |  |  |  |
| **Combined Safety** | ACENOCOUMAROL | 306 | 7.50 | 1 |  |
|  | Dabigatran | 257 | 6.60 | 0.88 | 0.74 – 1.04 |
|  |  |  |  |  |  |
| Gastrointestinal bleeding | ACENOCOUMAROL | 148 | 3.60 | 1 |  |
|  | Dabigatran | 192 | 4.90 | 1.36 | 1.09 – 1.70 |
|  |  |  |  |  |  |
| Intracranial bleeding | ACENOCOUMAROL | 158 | 3.80 | 1 |  |
|  | Dabigatran | 64 | 1.60 | 0.42 | 0.31 – 0.57 |

^1^ Incidence rates: number of events per 1,000 person-years of follow-up.

| **Supplementary Table 9: Acenocoumarol vs Rivaroxaban. Comparison of incidence density (/1,000 person-years of follow-up) by anticoagulant pairs according to event type. Number of pairs compared = 20,874 (8 years of follow-up between 2013-2020).** | | | | | |
| --- | --- | --- | --- | --- | --- |
|  | | | |  |  |
| **ACENOCOUMAROL - Rivaroxaban** | **ANTICOAGULANT** | **Events**  **(n)** | **Incidence Rates^1^** | **Relative**  **Risk** | **95%CI** |
| **Effectiveness** |  |  |  |  |  |
| **Combined Effectiveness** | ACENOCOUMAROL | 6,041 | 75.76 | 1 |  |
|  | Rivaroxaban | 4,522 | 64.47 | 0.85 | 0.82-0.88 |
|  |  |  |  |  |  |
| All-cause mortality | ACENOCOUMAROL | 4,822 | 60.47 | 1 |  |
|  | Rivaroxaban | 3,357 | 47.86 | 0.79 | 0.76-0.83 |
|  |  |  |  |  |  |
| Transient ischaemic attack | ACENOCOUMAROL | 464 | 5.82 | 1 |  |
|  | Rivaroxaban | 480 | 6.84 | 1.18 | 1.04-1.34 |
|  |  |  |  |  |  |
| Systemic embolism | ACENOCOUMAROL | 49 | 0.61 | 1 |  |
|  | Rivaroxaban | 36 | 0.51 | 0.84 | 0.54-1.28 |
|  |  |  |  |  |  |
| Pulmonary embolism | ACENOCOUMAROL | 79 | 0.99 | 1 |  |
|  | Rivaroxaban | 53 | 0.76 | 0.76 | 0.54-1.08 |
|  |  |  |  |  |  |
| Ischaemic stroke | ACENOCOUMAROL | 627 | 7.86 | 1 |  |
|  | Rivaroxaban | 597 | 8.51 | 1.08 | 0.97-1.21 |
|  |  |  |  |  |  |
| **Safety** |  |  |  |  |  |
| **Combined Safety** | ACENOCOUMAROL | 692 | 8.20 | 1 |  |
|  | Rivaroxaban | 441 | 6.00 | 0.73 | 0.64 – 0.82 |
|  |  |  |  |  |  |
| Gastrointestinal bleeding | ACENOCOUMAROL | 398 | 4.70 | 1 |  |
|  | Rivaroxaban | 292 | 4.00 | 0.84 | 0.72 – 0.98 |
|  |  |  |  |  |  |
| Intracranial bleeding | ACENOCOUMAROL | 294 | 3.50 | 1 |  |
|  | Rivaroxaban | 149 | 2.00 | 0.58 | 0.47 – 0.71 |

^1^ Incidence rates: number of events per 1,000 person-years of follow-up.

| **Supplementary Table 10: Acenocoumarol vs Apixaban. Comparison of incidence density (/1,000 person-years of follow-up) by anticoagulant pairs according to event type. Number of pairs compared = 18,431 (7 years of follow-up between 2014-2020).** | | | | | |
| --- | --- | --- | --- | --- | --- |
|  | | |  |  |  |
| **ACENOCOUMAROL - Apixaban** | **ANTICOAGULANT** | **Events**  **(n)** | **Incidence Rates^1^** | **Relative**  **Risk** | **95%CI** |
| **Effectiveness** |  |  |  |  |  |
| **Combined Effectiveness** | ACENOCOUMAROL | 5,594 | 94.15 | 1 |  |
|  | Apixaban | 3,747 | 82.42 | 0.88 | 0.84-0.91 |
|  |  |  |  |  |  |
| All-cause mortality | ACENOCOUMAROL | 4,515 | 75.99 | 1 |  |
|  | Apixaban | 2,927 | 64.38 | 0.85 | 0.81-0.89 |
|  |  |  |  |  |  |
| Transient ischaemic attack | ACENOCOUMAROL | 401 | 6.75 | 1 |  |
|  | Apixaban | 359 | 7.9 | 1.17 | 1.01-1.35 |
|  |  |  |  |  |  |
| Systemic embolism | ACENOCOUMAROL | 42 | 0.71 | 1 |  |
|  | Apixaban | 27 | 0.59 | 0.84 | 0.51-1.35 |
|  |  |  |  |  |  |
| Pulmonary embolism | ACENOCOUMAROL | 72 | 1.21 | 1 |  |
|  | Apixaban | 65 | 1.43 | 1.18 | 0.84-1.65 |
|  |  |  |  |  |  |
| Ischaemic stroke | ACENOCOUMAROL | 564 | 9.49 | 1 |  |
|  | Apixaban | 370 | 8.14 | 0.86 | 0.75-0.98 |
|  |  |  |  |  |  |
| **Safety** |  |  |  |  |  |
| **Combined Safety** | ACENOCOUMAROL | 549 | 8.70 | 1 |  |
|  | Apixaban | 360 | 7.40 | 0.85 | 0.74 – 0.97 |
|  |  |  |  |  |  |
| Gastrointestinal bleeding | ACENOCOUMAROL | 319 | 5.00 | 1 |  |
|  | Apixaban | 216 | 4.40 | 0.87 | 0.73 – 1.04 |
|  |  |  |  |  |  |
| Intracranial bleeding | ACENOCOUMAROL | 230 | 3.60 | 1 |  |
|  | Apixaban | 144 | 2.90 | 0.81 | 0.65 – 0.99 |

Incidence rates: number of events per 1,000 person-years of follow-up.

| **Supplementary Table 11: Acenocoumarol vs Edoxaban. Comparison of incidence density (/1,000 person-years of follow-up) by anticoagulant pairs according to event type. Number of pairs compared = 3,080 (4 years of follow-up between 2017-2020).** | | | | | |
| --- | --- | --- | --- | --- | --- |
|  | | | | | |
| **ACENOCOUMAROL - Edoxaban** | **ANTICOAGULANT** | **Events**  **(n)** | **Incidence Rates^1^** | **Relative**  **Risk** | **95%CI** |
| **Effectiveness** |  |  |  |  |  |
| **Combined Effectiveness** | ACENOCOUMAROL | 446 | 67.81 | 1 |  |
|  | Edoxaban | 312 | 64.12 | 0.95 | 0.82-1.09 |
|  |  |  |  |  |  |
| All-cause mortality | ACENOCOUMAROL | 342 | 52 | 1 |  |
|  | Edoxaban | 234 | 48.09 | 0.92 | 0.78-1.09 |
|  |  |  |  |  |  |
| Transient ischaemic attack | ACENOCOUMAROL | 39 | 5.93 | 1 |  |
|  | Edoxaban | 25 | 5.14 | 0.87 | 0.52-1.42 |
|  |  |  |  |  |  |
| Systemic embolism | ACENOCOUMAROL | 2 | 0.3 | 1 |  |
|  | Edoxaban | 5 | 1.03 | 3.38 | 0.73-23.6 |
|  |  |  |  |  |  |
| Pulmonary embolism | ACENOCOUMAROL | 11 | 1.67 | 1 |  |
|  | Edoxaban | 4 | 0.82 | 0.49 | 0.14-1.44 |
|  |  |  |  |  |  |
| Ischaemic stroke | ACENOCOUMAROL | 52 | 7.90 | 1 |  |
|  | Edoxaban | 44 | 9.04 | 1.14 | 0.76-1.71 |
|  |  |  |  |  |  |
| **Safety** |  |  |  |  |  |
| **Combined Safety** | ACENOCOUMAROL | 56 | 8.2 | 1 |  |
|  | Edoxaban | 30 | 5.9 | 0.71 | 0.44 – 1.13 |
|  |  |  |  |  |  |
| Gastrointestinal bleeding | ACENOCOUMAROL | 26 | 3.8 | 1 |  |
|  | Edoxaban | 23 | 4.5 | 1.18 | 0.64 – 2.15 |
|  |  |  |  |  |  |
| Intracranial bleeding | ACENOCOUMAROL | 30 | 4.4 | 1 |  |
|  | Edoxaban | 7 | 1.4 | 0.31 | 0.11 – 0.72 |

^1^ Incidence rates: number of events per 1,000 person-years of follow-up.

| **MATCHED GROUPS** | | **EFFECTIVENESS EVENTS^1^** | | |
| --- | --- | --- | --- | --- |
|  | **PATIENTS**  **(pairs)** | **EVENTS**  **(n)** | **Hazard Ratio (HR)** | **MULTIVARIATE**  **MODEL** |
| Warfarin | 14,942 (7,471) | 4,462 | 1.05 (0.99-1.12) | COX |
| Dabigatran | 19,424 (9,712) | 4,397 | 0.82 (0.77-0.87) | COX |
| Rivaroxaban | 41,748 (20,874) | 10,563 | 0.91 (0.88-0.95) | COX |
| Apixaban | 36,862 (18,431) | 9,341 | 0.96 (0.92-1.00) | COX |
| Edoxaban | 6,160 (3,080) | 758 | Curves do not meet criteria | Kaplan-Meier curves |
|  |  |  |  |  |
|  |  |  |  |  |
| **MATCHED GROUPS** | | **SAFETY EVENTS^2^** | | |
|  | **PATIENTS**  **(pairs)** | **EVENTS**  **(n)** | **Hazard Ratio (HR**) | **MULTIVARIATE**  **MODEL** |
| Warfarin | 14,942 (7,471) | 556 | 1.64 (1.38-1.95) | COX |
| Dabigatran | 19,424 (9,712) | 563 | 0.84 (0.71-0.99) | COX |
| Rivaroxaban | 41,748 (20,874) | 1,133 | 0.71 (0.63-0.80) | COX |
| Apixaban | 36,862 (18,431) | 909 | 0.87 (0.76-1.00) | COX |
| Edoxaban | 6,160 (3,080) | 86 | Curves do not meet criteria | Kaplan-Meier curves |
|  |  |  |  |  |
|  | |  |  |  |
| ^1^ Combined effectiveness: transient ischaemic attack, systemic embolism, pulmonary embolism, ischaemic stroke or all-cause mortality. | | | | |
| ^2^ Combined safety: gastrointestinal or intracranial bleeding. | | | | |

**Supplementary Table 12: Multivariate analysis between anticoagulant pairs after adjustment**

**for confounding variables. Comparator: Acenocoumarol.**
