# Supplementary material for: Comparative clinical outcomes of acenocoumarol versus direct oral anticoagulants (DOACs) and warfarin in patients with atrial fibrillation: real-world-evidence (SIESTA-A study)
Source: Front Pharmacol. 2025 Aug 1;16:1548298. doi: 10.3389/fphar.2025.1548298 (PMC12354484; doi:10.3389/fphar.2025.1548298)
Supplement: Supplementary file 2 [file DataSheet1.docx]

**Supplementary Figure 1**. Selection of the probability range (p) for matching patients undergoing treatment with: (A) acenocoumarol and warfarin (overlap range: 0.40-0.94); (B) acenocoumarol and dabigatran (overlap range: 0.45-0.94); (C) acenocoumarol and rivaroxaban (overlap range: 0.30-0.95); (D) acenocoumarol and apixaban (overlap: 0.30-0.90) and (E) acenocoumarol and edoxaban (overlap range: 0.45-0.90).

1.
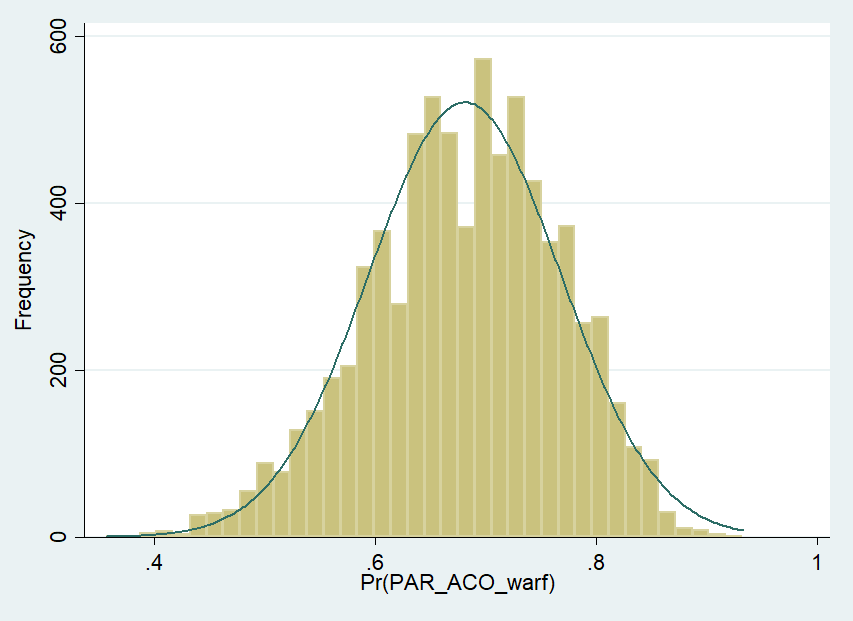

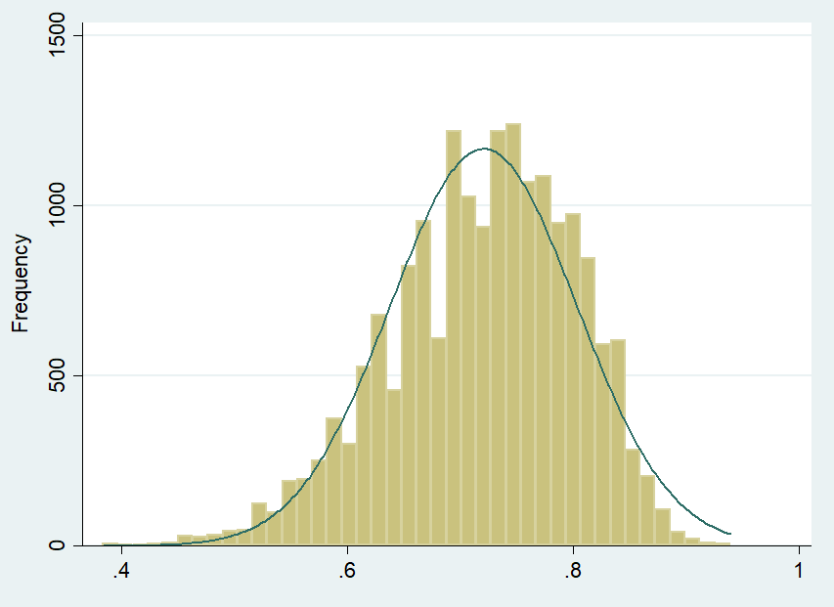
Acenocoumarol Warfarin

Probability

Probability

1.
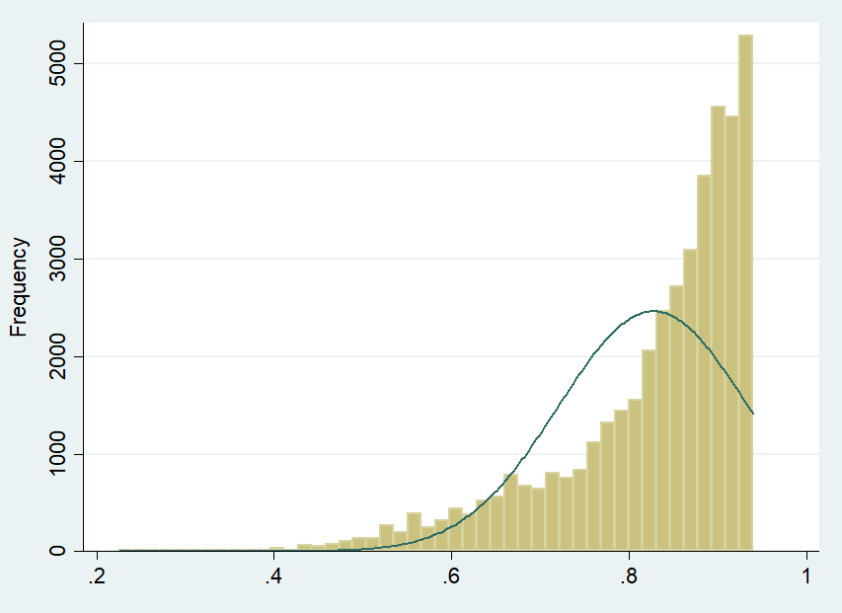

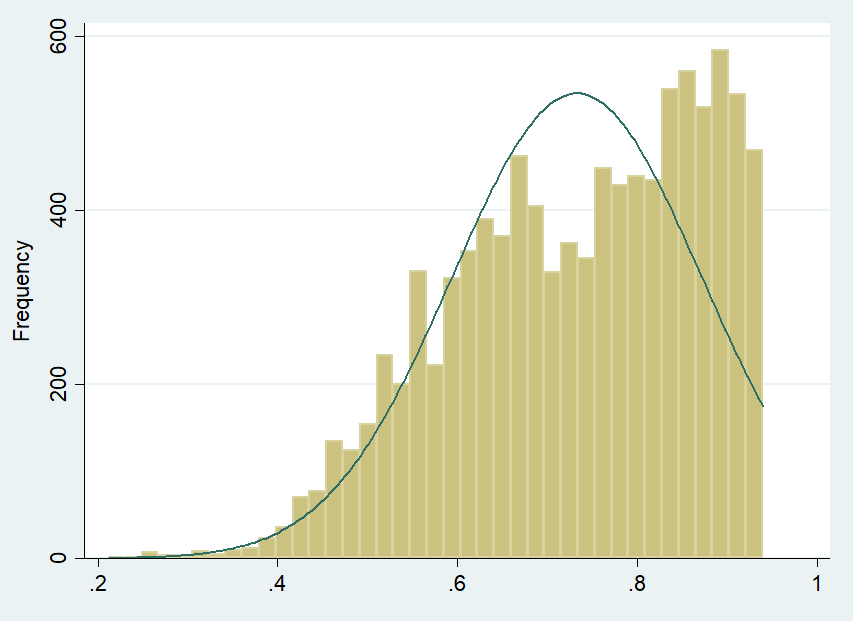
Acenocoumarol Dabigatran

Probability

Probability

1. Acenocoumarol Rivaroxaban


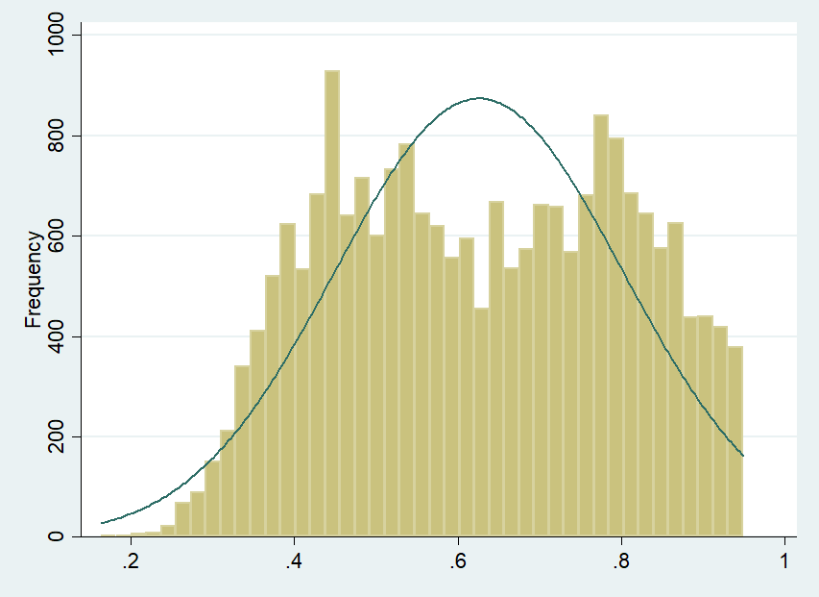


Probability


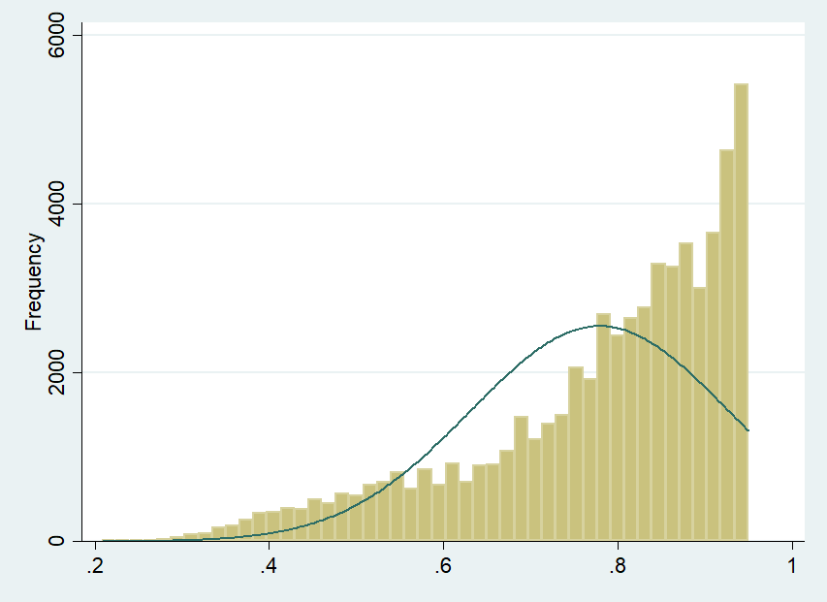


Probability

1. Acenocoumarol Apixaban


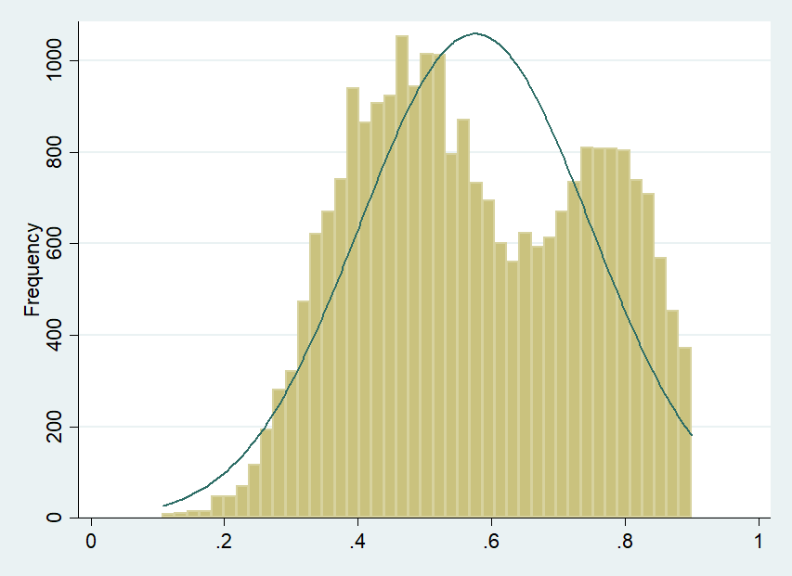


Probability


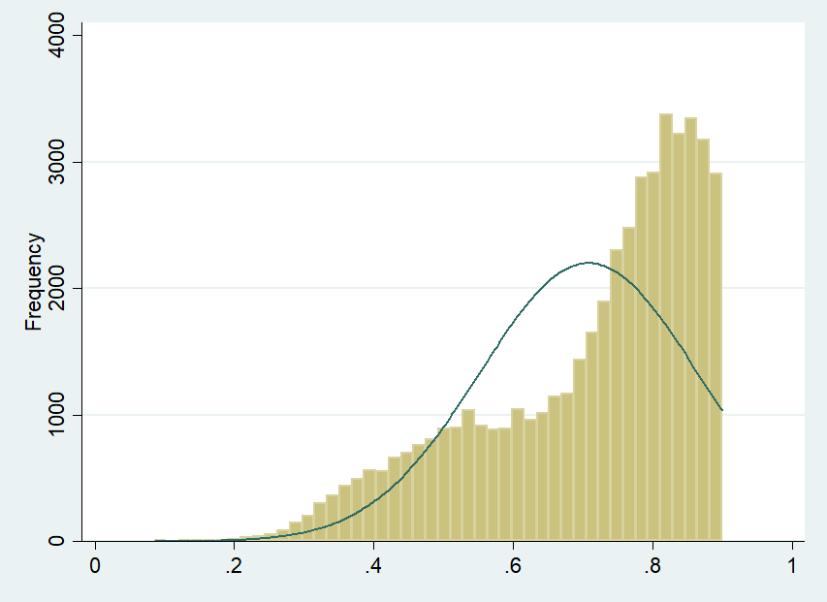


Probability

1. Acenocoumarol Edoxaban


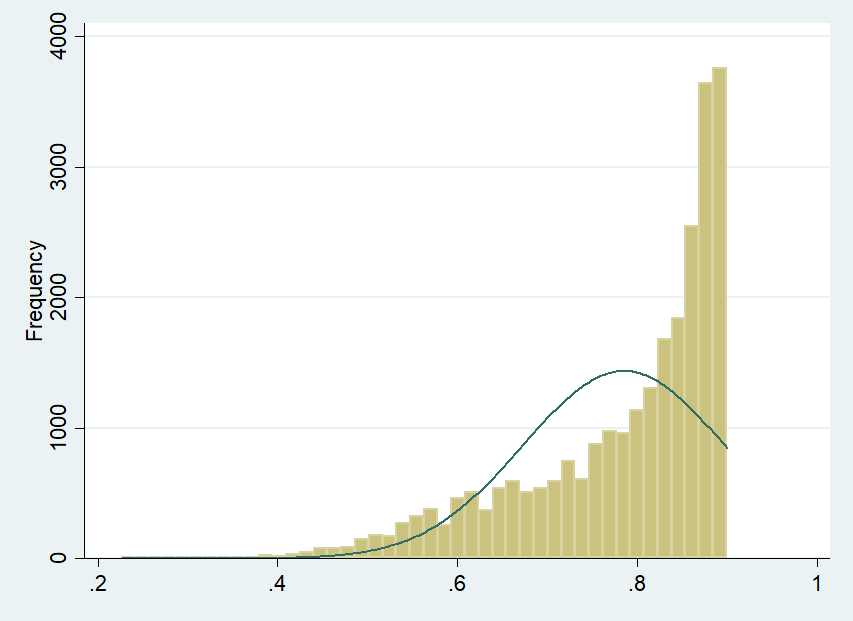


Probability


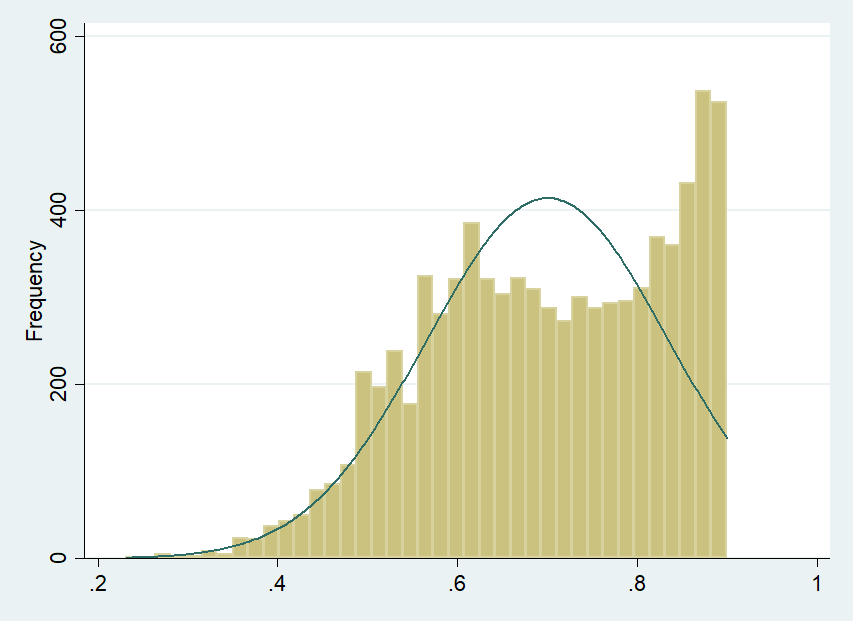


Probability
